# Enhanced Quantitative Phosphocreatine MR Imaging of Skeletal Muscle Using a Global–Local Two‐Branch Deep Learning Model

**DOI:** 10.1002/mrm.70386

**Published:** 2026-04-10

**Authors:** Malvika Viswanathan, Leqi Yin, Yashwant Kurmi, Shuyang Chai, Xiaoyu Jiang, Yuankai Huo, Junzhong Xu, Limin Chen, John C. Gore, Zhongliang Zu

**Affiliations:** ^1^ Vanderbilt University Institute of Imaging Science, Vanderbilt University Medical Center Nashville Tennessee USA; ^2^ Department of Biomedical Engineering Vanderbilt University Nashville Tennessee USA; ^3^ School of Engineering Vanderbilt University Nashville Tennessee USA; ^4^ Department of Radiology and Radiological Sciences Vanderbilt University Medical Center Nashville Tennessee USA; ^5^ Department of Electrical and Computer Engineering Vanderbilt University Nashville Tennessee USA; ^6^ Department of Computer Science Vanderbilt University Nashville Tennessee USA; ^7^ Department of Physics and Astronomy Vanderbilt University Nashville Tennessee USA; ^8^ Molecular Physiology and Biophysics Vanderbilt University Nashville Tennessee USA

**Keywords:** amyotrophic lateral sclerosis, chemical exchange saturation transfer, deep learning, phosphocreatine

## Abstract

**Purpose:**

Phosphocreatine (PCr) is an essential marker of muscle metabolism, and accurate quantification of its (*f*_s_) and its exchange rate (*k*_sw_) is essential for diagnosing various muscular and neuromuscular diseases. Although chemical exchange saturation transfer (CEST) MRI can detect the saturation transfer effect from PCr, quantification of the underlying PCr f_s_ and k_sw_, particularly at low fields, remains challenging due to significant overlapping confounding effects in tissues when using conventional fitting approaches. Deep learning (DL) presents a promising alternative, yet traditional DL models often struggle to capture subtle PCr‐specific variations induced by changes in *f*_s_ or *k*_sw_. Furthermore, these models are typically trained on either fully synthetic data, which may not adequately mimic tissues, or in vivo data which lack ground truth.

**Methods:**

This study introduces a global–local two‐branch DL model to effectively eliminate confounding effects and capture subtle variations in the PCr CEST effect. Furthermore, our model was trained on partially synthetic data that offers both simulation flexibility and fidelity. Model accuracy was evaluated by using both digital and physical phantoms, and the model was applied to skeletal muscle of healthy rats and rats with amyotrophic lateral sclerosis (ALS).

**Results:**

Phantom experiments demonstrate that our approach surpasses all fitting methods, the *state‐of‐the‐art* model, and other combinations of DL models and training data. In vivo, the model identified a significant reduction in PCr *f*_s_ in ALS rats, which other methods fail to detect.

**Conclusions:**

Our global–local two‐branch DL model trained using partially synthetic data enhances PCr quantification in skeletal muscle.

## 1 Introduction

Phosphocreatine (PCr) plays a critical role in muscle energy metabolism by serving as a rapid reserve of high‐energy phosphates [1]. Through the creatine kinase (CK) reaction, PCr donates a phosphate group to adenosine diphosphate (ADP) to regenerate adenosine triphosphate (ATP), the primary cellular energy carrier [2]. Changes in PCr concentration reflect alterations in CK activity and muscle metabolism and are associated with various muscular and neuromuscular diseases. Monitoring PCr therefore provides important insight into muscle energy status and CK system efficiency for both research and clinical applications. Traditionally, PCr levels have been assessed through blood assays; however, this approach is invasive and provides limited information about muscle‐specific metabolism [3]. Phosphorus‐31 magnetic resonance spectroscopy (^31^P MRS) offers a non‐invasive alternative for detecting PCr concentration, but its application is limited by low sensitivity, poor spatial resolution, and the requirement for specialized hardware [4].

PCr can be detected using chemical exchange saturation transfer (CEST) MRI, a molecular imaging technique that enhances detection sensitivity beyond conventional MRS [5, 6, 7, 8, 9, 10, 11, 12]. In CEST imaging, radiofrequency saturation is applied at the resonant frequencies of exchangeable solute protons, which subsequently exchange with water protons and reduce the observable water signal. Repeating this exchange during saturation results in an accumulated decrease in the water signal, enabling indirect but sensitive detection of solute molecules and their chemical environment. The PCr CEST effect is primarily observed at 2.6 ppm from water. Various fitting approaches, including multiple‐pool Lorentzian fitting (mfit) [13] and polynomial and Lorentzian line‐shape fitting (PLOF) [4, 13, 14, 15, 16, 17, 18, 19, 20, 21] have been used to isolate the PCr CEST effect from these other CEST effects on the Z‐spectrum. However, robust and accurate quantification of the underlying PCr solute water fraction (*f*_s_) and pH‐sensitive exchange rate (*k*_sw_), which are more closely related to CK reactions and pathologies, remains challenging and relatively understudied due to background confounding signals, particularly at relatively low magnetic fields. Additionally, subtle variations in the PCr CEST effect caused by changes in either PCr *f*_s_ or *k*_sw_ further exacerbate the challenge in robust and accurate quantification.

Deep learning (DL) methods have recently shown strong potential for CEST MRI analysis and quantification of individual CEST effects [22, 23, 24, 25, 26, 27, 28, 29, 30, 31, 32, 33, 34, 35, 36, 37, 38, 39, 40]. For example, the *state‐of‐the‐art* (*SOTA*) DL approach using a fully connected neural network model, termed ANNCEST, has been employed to quantify PCr *f*_s_ and *k*_sw_ in muscle tissues, demonstrating significant improvements over traditional fitting methods [15]. Nevertheless, challenges remain under low signal‐to‐noise ratio (SNR), which is common in muscle imaging. Conventional DL architectures often capture global spectral patterns but may struggle to detect subtle local variations in the PCr CEST effect associated with changes by variations in PCr *f*_s_ and *k*_sw_, leading to potential inaccuracies in quantification. Moreover, DL models for CEST MRI are typically trained using two primary types of data: in vivo and fully synthetic datasets. In vivo data, derived from biological samples, offer realistic tissue representations. However, they often suffer from insufficient training data and lack ground truth. Conversely, fully synthetic data, generated by simulating various sample parameters, may provide sufficient training data and ground truth, but accurately modeling tissues with synthetic datasets requires comprehensive prior knowledge of tissue composition. Addressing all these challenges requires refining DL models to enhance sensitivity to subtle CEST effects and improving synthetic data generation to better mimic real tissue characteristics.

We developed a two‐branch DL architecture to improve PCr quantification. This architecture comprises a global branch module and a local branch module. The global branch module is tasked with capturing background confounding signals along with the noise distribution across the entire Z‐spectrum, while the local branch module is dedicated to detecting subtle variations in the PCr CEST effect within a specific section of the Z‐spectrum. This global–local two‐branch approach balances the extraction of both global and local features, ensuring the subtle variations in PCr effects are accurately captured. Furthermore, we employed partially synthetic training data that offers greater fidelity than fully synthetic datasets while retaining the simulation flexibility and providing ground truth data. Instead of merely mixing in vivo and simulated data, we integrate the measured line‐shape information of CEST effects that are difficult to model analytically. These measured line shapes are incorporated into a simulation framework, allowing us to vary their relative ratios alongside the *f*_s_ and *k*_sw_ values of CEST components that are easier to simulate. This approach enables us to generate data that not only retain a well‐defined ground truth but also closely replicate the characteristics of real in vivo measurements, thereby improving both realism and diversity in the training dataset.

Previously, we developed a method for generating partially synthetic data for APT quantification [41, 42]. In this study, we adapted and extended this approach for PCr quantification. Our method was initially validated on both digital and physical phantoms to assess its accuracy and robustness against several traditional fitting methods and the *SOTA* method [15]. Subsequently, it was applied in an animal study involving both healthy rats and those with amyotrophic lateral sclerosis (ALS).

## 2 Methods

### 2.1 MRI

Experiments were conducted on a Varian DirectDrive horizontal 4.7 T MRI system equipped with a 38 mm Doty RF coil (Doty Scientific Inc., Columbia, SC, USA). Two cohorts of animals were imaged in this study. The first cohort was used for repeatability analysis and consisted of eight Wild type (Wt.) with both right and left hind limbs imaged for each animal. The second cohort included Wt and tSOD1G93A ALS transgenic rats aged greater than 200 days. Animals were anesthetized with 2% isoflurane in 98% oxygen and positioned for hindlimb imaging. Physiological parameters were monitored throughout the experiment, including respiration and rectal temperature, which was maintained at 37°C using a warm‐air feedback system (SA Instruments, Stony Brook, NY, USA). CEST images were acquired with Δ*ω* ranging from ±10 to ±5 ppm in 1.25 ppm intervals, and −5 to 5 ppm in 0.125 ppm intervals. Control images were acquired with Δ*ω* set at 500 ppm. The CEST sequence consisted of a 5 s continuous wave (CW) rectangular saturation pulse at B_1_ of 0.5μT or 1μT, followed by data acquisition and a 2 s recovery period. The total scan time for acquiring the CEST data at both B_1_ values was 21mins 16 s with a single average. The observed water longitudinal relaxation time (*T*_1obs_ = 1/*R*_1obs_) *R*_1obs_ and MT pool size ratio (*f*_m_) images were determined using a selective inversion recovery method [43]. All images were acquired using single‐shot spin‐echo echo planar imaging (SE‐EPI), with a matrix size of 64 × 64, a field of view of 35 mm × 35 mm and a slice thickness of 2 mm. All animal experiments were conducted under protocols approved by the Vanderbilt University Medical Center Institutional Animal Care and Use Committee (M2000039‐01 approved 05/10/2023).

### 2.2 Traditional Fitting Approaches and *SOTA* Model for PCr Quantification

For comparisons, the mfit, PLOF, and the *SOTA* model were employed. In the mfit method, each CEST effect is modeled using a Lorentzian function, defined as $L\left(\Delta \omega \right)=\frac{A}{1+\frac{{\left(\Delta \omega -\Delta \right)}^{2}}{{\left(0.5\Gamma \right)}^{2}}}$ with A representing the amplitude and Γ representing the spectral width of the effect. For N number of CEST effects, the Z‐spectrum can be modeled as the sum of all Lorentzian functions:

(1) $$S\left(\Delta \omega \right)=1-\sum_{i=1}^{N}L_{i}\left(\Delta \omega \right)$$

A six‐pool model (*N* = 6) (APT, PCr, amines/guanidine, water, NOE, and MT) was employed consistent with the previous study [13, 44]. The initial and boundary conditions for the Lorentzian fitting process in this study are given in Table S1. After applying mfit fitting, the reference signal (*S*_ref_) was generated by reconstructing the fitted signal with the amplitude of the PCr CEST effect set to zero (i.e., A = 0 when Δω=Δ), while the label signal (*S*_lab_) was directly taken from the measured CEST data. The PCr CEST effect was quantified using an apparent exchange‐dependent relaxation (AREX) metric, which subtracts 1/S_lab_ from 1/*S*_ref_, and normalizing by *T*_1obs [_ 45, 46_]_. In the PLOF method, PCr and guanidine CEST effects were modeled using two Lorentzian functions, while broad background contributions were modeled with a third‐order polynomial. The Z‐spectrum was expressed as the inverse sum of these effects, and the PCr CEST effect was extracted from the Lorentzian component. Both methods used median filtering (medfilt1, size = 3) and least‐squares optimization. To further quantify the values of PCr *f*_s_ and *k*_sw_, the fitted PCr CEST effects with Δ*ω* ranging from −10 to 10 ppm, at the two acquired B_1_ values, were fitted to [2],

(2) $$R_{\mathrm{ex}}\left(\Delta \omega \right)=\frac{f_{s}k_{\mathrm{sw}}\omega_{1}^{2}}{\omega_{1}^{2}+\left(R_{2s}+k_{\mathrm{sw}}\right)k_{\mathrm{sw}}+\frac{{\left(\Delta \omega -\Delta \right)}^{2}k_{\mathrm{sw}}}{R_{2s}+k_{\mathrm{sw}}}}$$

where *R*_ex_ represents the AREX‐PCr CEST effect quantified either with mfit or PLOF method; R_2s_ denotes the solute transverse relaxation rate; $\omega_{1}=\gamma B_{1}$, where γ is the gyromagnetic ratio of proton. The previous *SOTA* (ANNCEST) model used a single Z‐spectrum at B_1_ = 0.6μT and with Δ*ω* ranging from 0.5 to 4 ppm as inputs. In this study, the same *SOTA* architecture was applied, using a single Z‐spectrum at B_1_ of 0.5μT while maintaining the same Δ*ω* range as input.

### 2.3 Generation of Partially Synthetic Data

**FIGURE 1:**
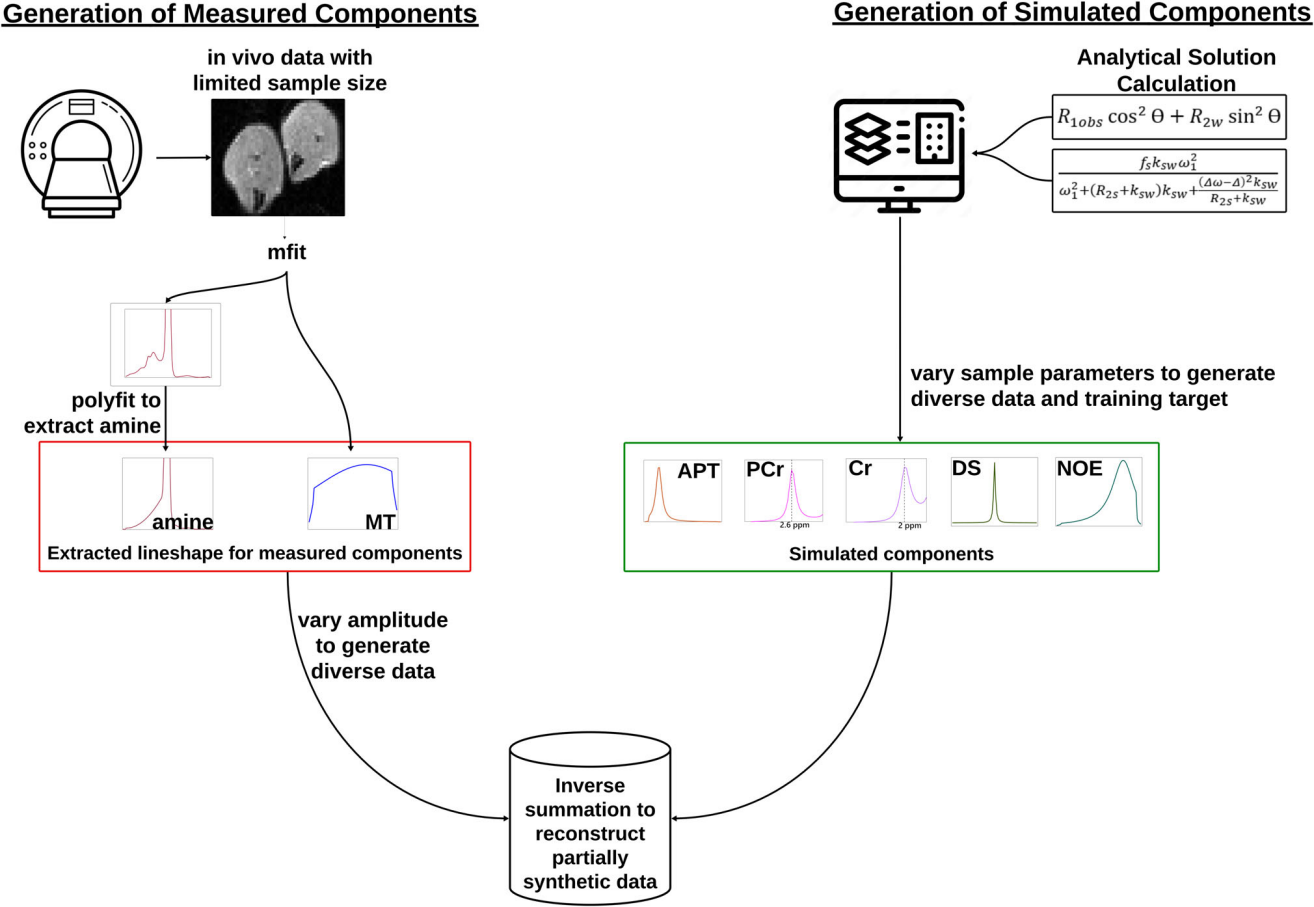
Schematic illustrating the generation of partially synthetic training data.

CEST signals can be described as the ratio of *R*_1obs_ to the sum of DS, MT, and all CEST effects [45, 46]. To generate partially synthetic CEST signals, we categorize the components into two types: measured components and simulated components. Measured components, which involve pools with significant overlap, unknown sample parameter range, or complex behaviors that are challenging to simulate, are obtained through the mfit method. Simulated components, featuring simple signal models, are obtained through analytical calculations. By combining these components using the inverse summation, CEST signals with greater fidelity than those from fully synthetic datasets can be reconstructed, while retaining the simulation flexibility. Figure 1 illustrates the method to generate the partially synthetic data, encompassing the generation of measured components and simulated components and the inverse summation for reconstructing CEST signals. Equation (3) presents the inverse summation formula used to generate the partially synthetic CEST data.

(3) $$\frac{S\left(\Delta \omega \right)}{S_{0}}=\frac{R_{1\mathrm{obs}}}{\begin{array}{ll} & R_{\mathrm{eff}}\left(\Delta \omega \right)+\frac{R_{\mathrm{ex}}^{\mathrm{APT}}\left(\Delta \omega \right)}{1+r_{\mathrm{MT}}f_{m}}+\frac{R_{\mathrm{ex}}^{\mathrm{PCr}}\left(\Delta \omega \right)}{1+r_{\mathrm{MT}}f_{m}}+\frac{R_{\mathrm{ex}}^{\text{guan}}\left(\Delta \omega \right)}{1+r_{\mathrm{MT}}f_{m}}\\ & +\frac{R_{\mathrm{ex}}^{\mathrm{NOE}}\left(\Delta \omega \right)}{1+r_{\mathrm{MT}}f_{m}}+r_{\text{amines}}\frac{R_{\mathrm{ex}}^{\text{amines}}\left(\Delta \omega \right)}{1+r_{\mathrm{MT}}f_{m}}+r_{\mathrm{MT}}R_{\mathrm{ex}}^{\mathrm{MT}}\left(\Delta \omega \right) \end{array}}\frac{\Delta \omega^{2}}{\omega_{1}^{2}+\Delta \omega^{2}}$$

(4) $$R_{\mathrm{eff}}\left(\Delta \omega \right)=R_{1\mathrm{obs}}\frac{\Delta \omega^{2}}{\omega_{1}^{2}+\Delta \omega^{2}}+R_{2w}\frac{\omega_{1}^{2}}{\omega_{1}^{2}+\Delta \omega^{2}}$$

where the simulated components include $R_{\mathrm{eff}}$, $R_{\mathrm{ex}}^{\mathrm{APT}}$, $R_{\mathrm{ex}}^{\mathrm{PCr}}$, $R_{\mathrm{ex}}^{\text{guan}}$ and $R_{\mathrm{ex}}^{\mathrm{NOE}}$, representing the DS, APT, PCr CEST, guanidine CEST, and NOE effects, respectively. $R_{\mathrm{eff}}$ can be calculated using Equation (4), and R_ex_ effects for other simulated components can be calculated using Equation (2). The measured components, include $R_{\mathrm{ex}}^{\mathrm{MT}}$ and $R_{\mathrm{ex}}^{\text{amines}}$, representing the MT and amine CEST effects, respectively. $R_{\mathrm{ex}}^{\mathrm{MT}}$ can be obtained from the mfit method, and calculated using *R*_1obs_L_MT_/(1‐L_MT_) [47], where L_MT_ represents the direct subtraction of *S*_lab_ from *S*_ref_ (i.e., A_MT_ = 0 when $\Delta \omega =\Delta_{\mathrm{MT}}$) for the MT effect within the mfit method. *R*_1obs_ was calculated using the formula (*R*_1w_ + r_MT_ *f*_m_ *R*_1M_)/(1 + r_MT_ *f*_m_), where $R_{1M}$ is the MT pool longitudinal relaxation rate, and $f_{m}$, the MT pool concentration, can be measured directly. $R_{\mathrm{ex}}^{\text{amines}}$ can be obtained through a two‐step process. First, the combined effect $R_{\mathrm{ex}}^{\text{amines}/\text{guan}}$ which includes contributions from both amines and guanidine was obtained from the mfit method. Second, $R_{\mathrm{ex}}^{\text{amines}}$ was estimated by fitting $R_{\mathrm{ex}}^{\text{amines}/\text{guan}}$ in the 1–1.5 ppm and 3–5 ppm ranges to a third‐order polynomial function and extracting the baseline polynomial coefficients C_0_ to C_3_. $R_{\mathrm{ex}}^{\text{amines}}$ was defined as follows:

(5) $$\begin{array}{cc} R_{\mathrm{ex}}^{\text{amines}} & =C_{0}+C_{1}\left(\Delta \omega -\Delta_{\text{amine}}\right)+C_{2}{\left(\Delta \omega -\Delta_{\text{amine}}\right)}^{2}\\ & \;+C_{3}{\left(\Delta \omega -\Delta_{\text{amine}}\right)}^{3} \end{array}$$

where $\Delta_{\text{amine}}$ = 3 ppm. The initial and boundary conditions for this polynomial fitting process are given in Table S2. The measured components can be derived from a limited sample size to address the issue of insufficient in vivo training data, while maintaining diversity by adjusting its amplitude using a scaling factor (*r*_amines_ and r_MT_). The line shape information of the measured components does not require modification. It roughly reflects intrinsic molecular properties and thus exhibits relatively minor variation within specific tissues. In this study, measured components used to generate partially synthetic training data were obtained from a single Z‐spectrum of the digital phantom, a single physical phantom, or the average of 252 samples from one rat for the digital phantom, physical phantom, and animal studies, respectively. A wide range of training data was generated by systematically varying the sample parameters of each simulated component, including their *f*_s_, *k*_sw_, and *T*_2s_, along with *r*_amines_ and r_MT_ and water relaxations (*R*_1w_ and *R*_2w_). Additionally, variations in B_0_ and B_1_ inhomogeneities were introduced to better replicate real‐world conditions. Specifically, a B_0_ shift (Δ*ω*_shift_) was applied by replacing Δ*ω* with Δ*ω* + Δ*ω*_shift_, and a B_1_ shift (*β*) was applied by replacing *ω*_1_ with *βω*_1_ and replacing *r*_measured_ with *β* ^2^ *r*_measured [_ 4, 48_]_. The ground truth targets were the simulation parameters: PCr *f*_s_ and *k*_sw_. Details of generating partially synthetic data with measured components from digital phantoms and in vivo data are given in Table S3 while measured components taken from physical phantoms are given in Table S4.

### 2.4 Generation of Fully Synthetic Data and in Vivo Training Data

The fully synthetic data were generated through numerical simulations of Bloch equations using a seven‐pool model, which included amide, amine, PCr, guanidine, water, NOE and MT, representing major pools found in muscle tissue. A total of 26 572 050 samples were generated by varying the sample parameters, as well as including B_0_ and B_1_ shifts, as listed in Table S5. The ground truth targets were the simulation parameters: PCr *f*_s_ and *k*_sw_. For the in vivo training dataset, Z‐spectra were extracted from all voxels within the skeletal muscle of a single rat (252 samples). By applying B_0_ shifts, a dataset of 1260 samples was created. Additionally, an augmented dataset was generated by averaging neighboring voxel spectra, further increasing the dataset size to 793 170 samples. For the target data, the quantified PCr *f*_s_ and *k*_sw_ values using Equation (2) were used, with the PCr CEST effects fitted using either the mfit or PLOF methods.

### 2.5 Proposed DL Architecture and Training

**FIGURE 2:**
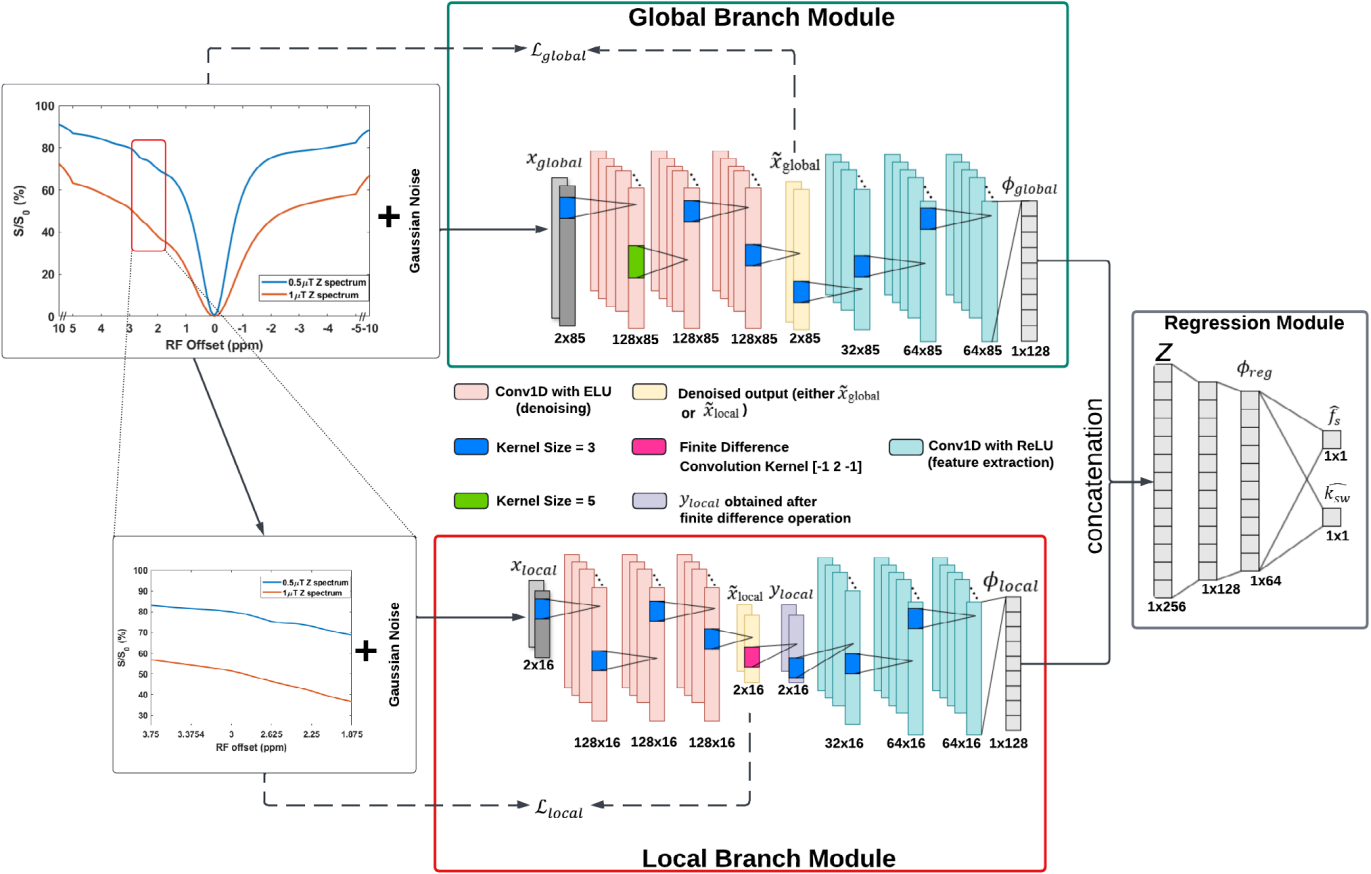
Architecture of the global–local two‐branch DL model designed to predict either *f*_s_ or *k*_sw_.

Figure 2 illustrates the architecture of the global–local two‐branch DL model, consisting of a global branch, a local branch, and a regression module. The global branch receives two full Z‐spectra at the acquired two B_1_ values, denoted as $x_{\text{global}}\in R^{2\times M}$, where M = 85 frequency offsets from 6.25 to −6.25 ppm were used. The local branch processes two spectral segments centered around the PCr region, denoted as $x_{\text{local}}\in R^{2\times M^{\prime }}$, where $M^{\prime }$ = 16 frequency offsets from 1.75 ppm to 3.75 ppm for the local branch were used. Both branches begin with three one‐dimensional convolutional layers (128 filters, ELU activation), dedicated to denoising [49]. The denoised outputs $\tilde{x}$_global_ and $\tilde{x}$_local_ are then passed to feature extraction layers. Within the global branch module, $\tilde{x}$_global_ is processed through three sequential convolutional layers (32, 64, and 64 filters, ReLU activation), followed by adaptive average pooling and a fully connected layer with 128 nodes to produce a compact feature representation $\phi_{\text{global}}\;\epsilon \;R^{128}$. Within the local branch module, $\tilde{x}$_local_ is first refined using a finite difference operator (*y*_local_), as defined in Equation (6) which is designed to approximate the second‐order derivative, along with zero padding to maintain the input's length.

(6) $$y_{\text{local}}\left(i\right)={\tilde{x}}_{\text{local}}\left(i-1\right)-2{\tilde{x}}_{\text{local}}\left(i\right)+{\tilde{x}}_{\text{local}}\left(i+1\right)\;\text{where}\;i=2,3,\ldots M^{\prime }-1$$

This operation enhances sensitivity to subtle spectral variations in the PCr region. The refined signal is then processed by convolutional feature extraction layers (32, 64, and 64 filters, ReLU activation), pooling, and a fully connected layer (128 nodes) to generate the local representation $\phi_{\text{local}}\;\epsilon \;R^{128}$. Finally, the regression module concatenates the global and local features to form $z\;\epsilon \;R^{256}$. This combined feature space is then processed through two fully connected layers with 128 and 64 nodes, culminating into two output heads that deliver the prediction output $\hat{f_{s}}$ and $\hat{k_{\mathrm{sw}}}$, which quantify both the PCr *f*_s_ and *k*_sw_.

All DL models were implemented in Python using the PyTorch library (version 2.5.1 with CUDA 12.4 support) trained using the Adam optimizer. Training was supervised using a composite loss consisting of a regression loss for predicting *f*_s_ and *k*_sw_ and denoising losses for the global and local branches:

(7) $$\begin{array}{cc} L_{\text{total}} & =\lambda_{\text{regress}}\times \left(\mathrm{MSE}\left(\hat{f_{s}},f_{s}\right)+\mathrm{MSE}\left(\hat{k_{\mathrm{sw}}},k_{\mathrm{sw}}\right)\right.\\ & \;\left.+\;\mathrm{MSE}\left(\hat{f_{s}}\hat{k_{\mathrm{sw}}},f_{s}k_{\mathrm{sw}}\right)\right)+\lambda_{\text{denoise}}\left(L_{\text{global}}+L_{\text{local}}\right) \end{array}$$

where $L_{\text{global}}$ is the mean squared error (MSE) between denoised global output $\tilde{x}$_global_ and clean global input, while $L_{\text{local}}$ is the MSE between denoised local output $\tilde{x}$_local_ and clean local input, across all batch samples. The dataset was divided into training and validation sets (80:20) and Z‐spectra were normalized by *R*_1obs_ prior to training, consistent with the AREX metric, where this normalization is essential to eliminate the influence of variations in water longitudinal relaxation. During training, Gaussian noise was dynamically added to each Z‐spectrum from the partially and fully synthetic data, with the standard deviation (*σ*) randomly chosen within the range of 0.001 to 0.04 for each epoch. Additional training details, network parameters, normalization procedures, and computational settings are provided in Table S6.

### 2.6 Experiments

To evaluate the proposed method, experiments were conducted using digital phantoms, physical phantoms, and in vivo animal data.

Digital phantoms and corresponding ground truth data were generated using numerical simulations of Bloch equations following the same pipeline as the fully synthetic data but with shifted sample parameters as listed in Table S7, to account for potential biases that may occur when modeling real tissue. Specifically, within each phantom, the PCr *f*_s_ and *k*_sw_ values were maintained constantly across voxels, while other sample parameters were varied to simulate complex tissue properties. A total of six digital phantoms were created: three with different *f*_s_ values and three with different *k*_sw_ values.

Nine physical phantoms were prepared in 1× phosphate‐buffered saline (PBS) each containing 5% bovine serum albumin (BSA), 50 mM creatine (Cr), and PCr concentrations of 50, 100, or 150 mM, with pH values of 6.8, 7.2, or 7.6. Z‐spectra from the phantom containing 100 mM PCr at pH 7.2 were used to extract measured components, while the remaining phantoms were used for testing. Additional PCr‐only phantoms with the same concentrations and pH values were prepared as ground truth. Ground truth f_s_ and k_sw_ were determined using a two‐step procedure. First, a three‐pool mfit model (water, PCr at 2.6 ppm, and PCr at 2 ppm) was applied to the PCr only phantoms at both B_1_ values to obtain the AREX‐PCr spectra. The initial and boundary conditions for the three pool mfit are given in Table S8. The resulting AREX spectra were truncated from 3 to 2.25 ppm, concatenated, and fitted to Equation (2) using least‐squares optimization to obtain the *f*_s_ and *k*_sw_.

1. Ablation Study: Seven distinct DL models (M_1_–M_7_), each with different combinations of the modules, were independently trained on the partially synthetic dataset and applied to the digital phantoms at SNRs of 40 dB, 33.98 and 30.46 dB. Unless otherwise stated, all models were trained using Z‐spectra at the two acquired B_1_ values. M_1_ includes only the regression module taking the concatenation of both entire Z‐spectra as input; M_2_ includes only the global branch module; M_3_ combines both the global branch and regression modules; M_4_ includes only the local branch module; M_5_ combines both the local branch and regression modules; M_6_ represents the complete model incorporating the global branch, local branch, and regression modules, trained using only a single Z‐spectrum at B_1_ = 0.5uT; M_7_ represents the complete model trained using Z‐spectra acquired at both B_1_ powers.
2. Comparative study: The proposed method (Two‐branch PS) was compared with traditional fitting methods (mfit and PLOF), the SOTA model trained on fully and partially synthetic data (SOTA FS and SOTA PS), and the two‐branch DL model trained on fully synthetic data (Two‐branch FS) using digital phantoms, physical phantoms, and in vivo data. For the in vivo analysis, additional models trained on in vivo data were also evaluated, including the two‐branch DL model trained using targets derived from mfit (DL_invivo_ mfit) and its augmented version (DL_invivo_ mfit (aug)), as well as models trained using targets derived from PLOF (DL_invivo_ PLOF) and its augmented version (DL_invivo_ PLOF (aug)). To assess regional variations, ROI‐averaged values were calculated from two anatomically distinct skeletal muscle regions: the posterior compartment (gastrocnemius–soleus) and the anterior compartment (tibialis anterior and extensor muscles).
3. To assess repeatability, contralateral left–right comparisons were performed in the first cohort of eight Wt Animals. Whole‐muscle ROIs were defined for both hindlimbs using identical anatomical criteria. ROI‐averaged values for *f*_s_ and *k*_sw_ were extracted from each side, and paired left–right measurements were used to evaluate within‐animal consistency across quantification methods.

### 2.7 Statistics

Relative errors between predicted and ground truth values were calculated for all voxels in the digital phantoms and visualized using violin plots. Model accuracy was evaluated using the mean relative error (MRE) across phantoms with varied PCr *f*_s_ or *k*_sw_ values. Regions of interest (ROIs) for muscle tissue were identified based on *R*_1obs_ maps. Statistical comparison between the healthy and ALS groups was performed using Welch's *t*‐test and *p*‐value less than 0.05 was considered statistically significant. All data analysis was conducted using *MATLAB R2024b*.

## 3 Results

### 3.1 Ablation Study for the Two‐Branch Global–Local Model

**FIGURE 3:**
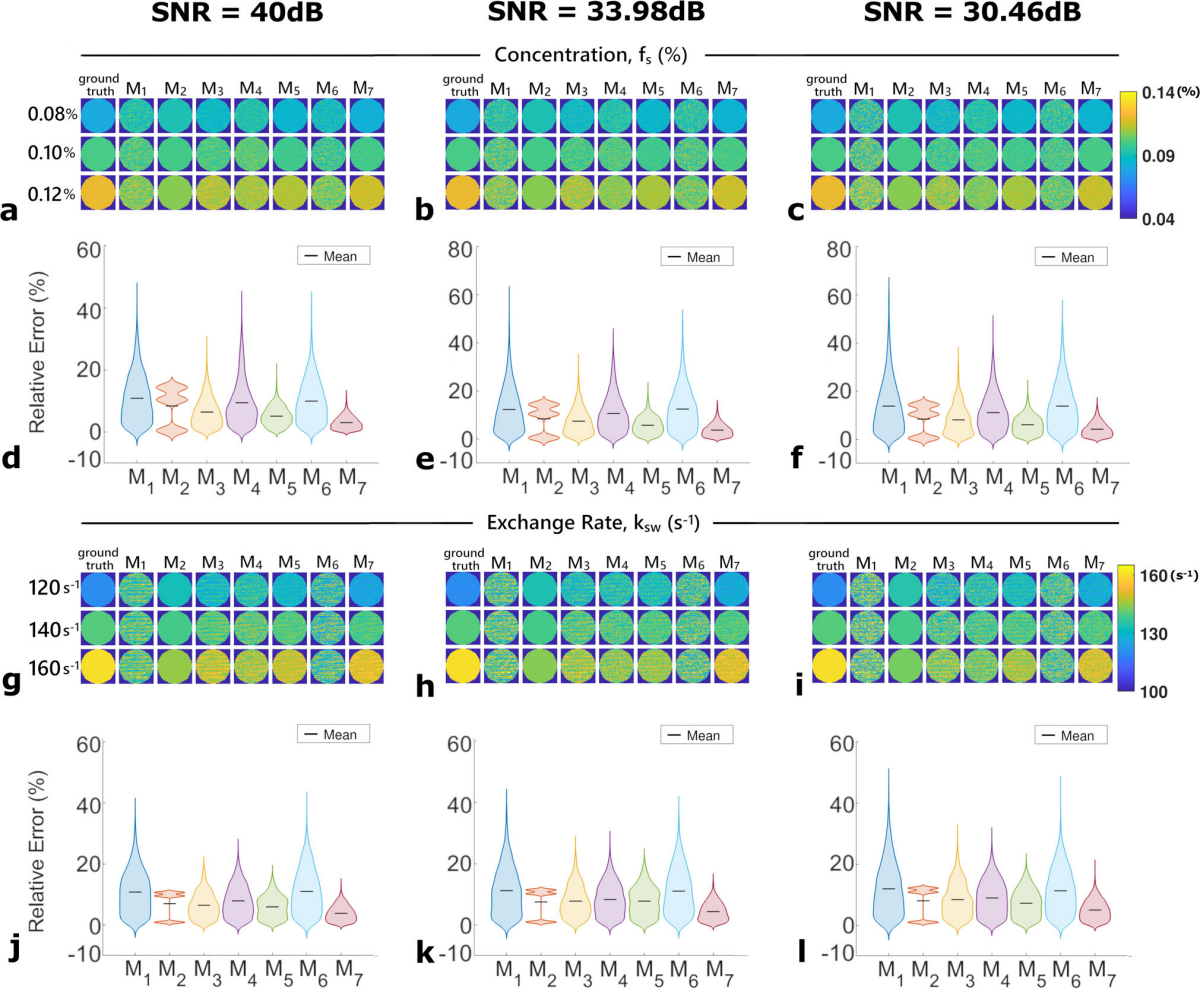
Ablation study evaluating the six model variants (M_1_–M_7_) for *f*_s_ and *k*_sw_ estimation under different SNR levels. (a–c) Digital phantoms displaying ground‐truth and predicted *f*_s_ maps corresponding to each noise level, obtained using various model variants. (d–f) Corresponding violin plots of the relative errors for *f*_s_. (g–i) Digital phantoms displaying ground‐truth and predicted *k*_sw_ maps corresponding to each noise level, obtained using various model variants. (j–l) Corresponding violin plots of the relative errors for *k*_sw_.

Figure 3 presents the ablation study results evaluating the performance of seven model variants (M_1_–M_7_) for predicting *f*_s_ and *k*_sw_ in the digital phantoms across various SNR levels. Figure 3a–c show the ground‐truth phantoms and predicted *f*_s_ maps, while Figure 3d–f present violin plots of the corresponding relative errors for phantoms with different *f*_s_ values. Figure 3g–l show the same layout for *k*_sw_ predictions.

Among all models, the proposed two‐branch DL model (M_7_) achieves the lowest MRE values (e.g., 3.00% ± 2.04% for *f*_s_ and 3.84% ± 2.51% for *k*_sw_ at SNR of 40 dB) and the narrowest spread of the relative errors, thus indicating superior accuracy and reduced susceptibility to confounding effects. Its performance also remains robust across noise levels, with MREs below 5% for both parameters even at SNR of 30.48 dB (4.14% ± 2.89% for *f*_s_ and 4.96% ± 3.15% for *k*_sw_), showing strong noise tolerance.

All other ablation models (M_1_–M_5_) produced substantially higher errors than M_7_, demonstrating the benefit of combining the global branch, local branch, and regression modules. At lower SNR, the M_2_ model showed lower MRE values than the M_4_ model compared to higher SNR levels, indicating that the global input carries more noise‐resilient information, likely due to its broader contextual encoding compared to the locally focused input. Additionally, at higher SNR, the M_5_ model shows lower MRE values than the M_3_ model, highlighting the sensitivity of the local branch to subtle changes in the PCr CEST effect. Moreover, the M_1_ model yields a higher MRE value than the M_2_ and M_3_ models, underscoring the critical role of both the denoising and feature extraction. Lastly, in most cases, the M_2_ and M_4_ models with regression exhibit higher MRE values than the M_3_ and M_5_ models without regression, emphasizing the important role of the regression module. In contrast, the M_6_ model trained using a single power showed noticeably higher errors (*f*_s_: 9.99% ± 2.74%, *k*_sw_:10.98% ± 4.02% at SNR = 40 dB) than models M_2_–M_5_ and M_7_, suggesting that a single saturation power does not provide sufficient information to reliably estimate both *f*_s_ and *k*_sw_. This highlights the importance of using dual power Z spectra to decouple the effects of *f*_s_ and *k*_sw_.

### 3.2 Comparison Study of Various Quantification Methods on Digital Phantoms

**FIGURE 4:**
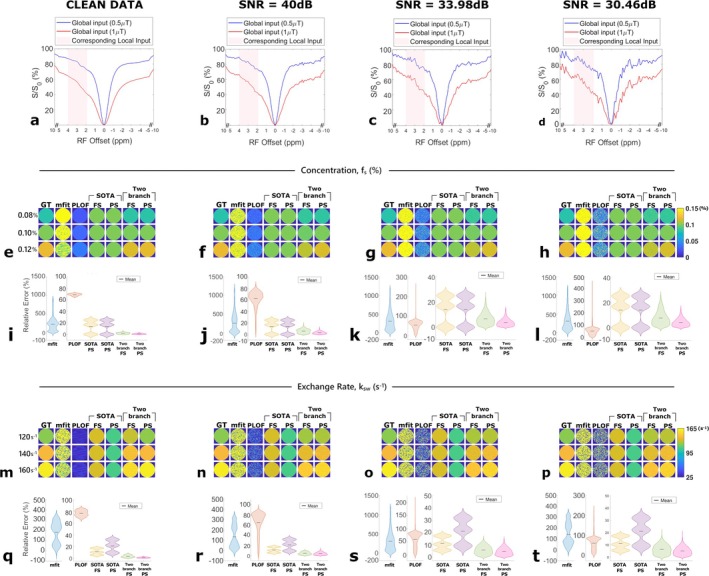
(a–d) Representative Z‐spectra at B_1_ of 0.5 and 1 μT for clean data and various noisy levels. (e–h) Digital phantoms displaying ground‐truth and predicted f_s_ maps corresponding to each noise level, obtained using six various quantification methods. (i–l) Corresponding violin plots of the relative errors for *f*_s_. (m–p) Digital phantoms displaying ground‐truth and predicted *k*_sw_ maps corresponding to each noise level, obtained using various quantification methods. (q–t) Corresponding violin plots of the relative errors for *k*_sw_. The six methods are multi‐pool Lorentzian fitting (mfit), polynomial and Lorentzian line‐shape fitting (PLOF), the *state‐of‐the‐art* (SOTA) model trained on fully synthetic (SOTA FS) and partially synthetic (SOTA PS) data, the proposed two‐branch global–local model trained on fully synthetic (Two branch FS) and partially synthetic (Two branch PS) data.

Figure 4 compares the performance of different methods for quantifying PCr *f*_s_ and *k*_sw_ on the digital phantoms across different SNR levels. Figure 4a–d shows the representative global and local input Z‐spectra at two B_1_ values from digital phantoms, while Figure 4e–h,m–p depict the ground truth digital phantoms and the corresponding quantification results for *f*_s_ and *k*_sw_, respectively. Violin plots in Figure 4i–l,q–t display the relative errors for various models in quantifying *f*_s_ and *k*_sw_, respectively.

Among all approaches, the proposed Two‐branch PS model achieved the MRE and the narrowest error distribution (e.g., 3.00% ± 2.04% for *f*_s_ and 3.84% ± 2.51% for *k*_sw_ at SNR = 40 dB), indicating the best performance. In contrast, traditional fitting methods showed substantially higher errors. For example, mfit produced MRE values of 257.14% ± 238.21% for *f*_s_ and 133.13% ± 107.19% for *k*_sw_, while PLOF yielded 63.02% ± 12.76% for *f*_s_ and 65% ± 21.12% for *k*_sw_ at SNR = 40 dB.

The *SOTA* models (SOTA FS, SOTA PS) offer reduced MRE values compared to the traditional fitting methods, yet the predicted *f*_s_ or *k*_sw_ values remain largely unchanged across all phantoms and all noise levels. This likely indicates the limited capacity of *SOTA* to learn the complex spectral patterns necessary for precise quantification. The Two‐branch FS model achieves reduced MRE values but not lower than the Two‐branch PS model, highlighting the advantages of utilizing partially synthetic data for training. Compared with the SOTA FS and SOTA PS models, the Two‐branch PS decreases the MRE values for *f*_s_ by 74.82% and 74.80%, and for *k*_sw_ by 65.12% and 81.70%, respectively. Remarkably, even at the lowest SNR level of 33.34, the Two‐branch PS model maintains MRE values below 5% for both parameters, whereas all other methods, particularly traditional ones, show increased error and variability. These findings underscore the superior accuracy and robustness of the Two‐branch PS model, demonstrating its efficacy in overcoming challenges.

### 3.3 Comparison Study of Various Quantification Methods in Physical Phantoms

**FIGURE 5:**
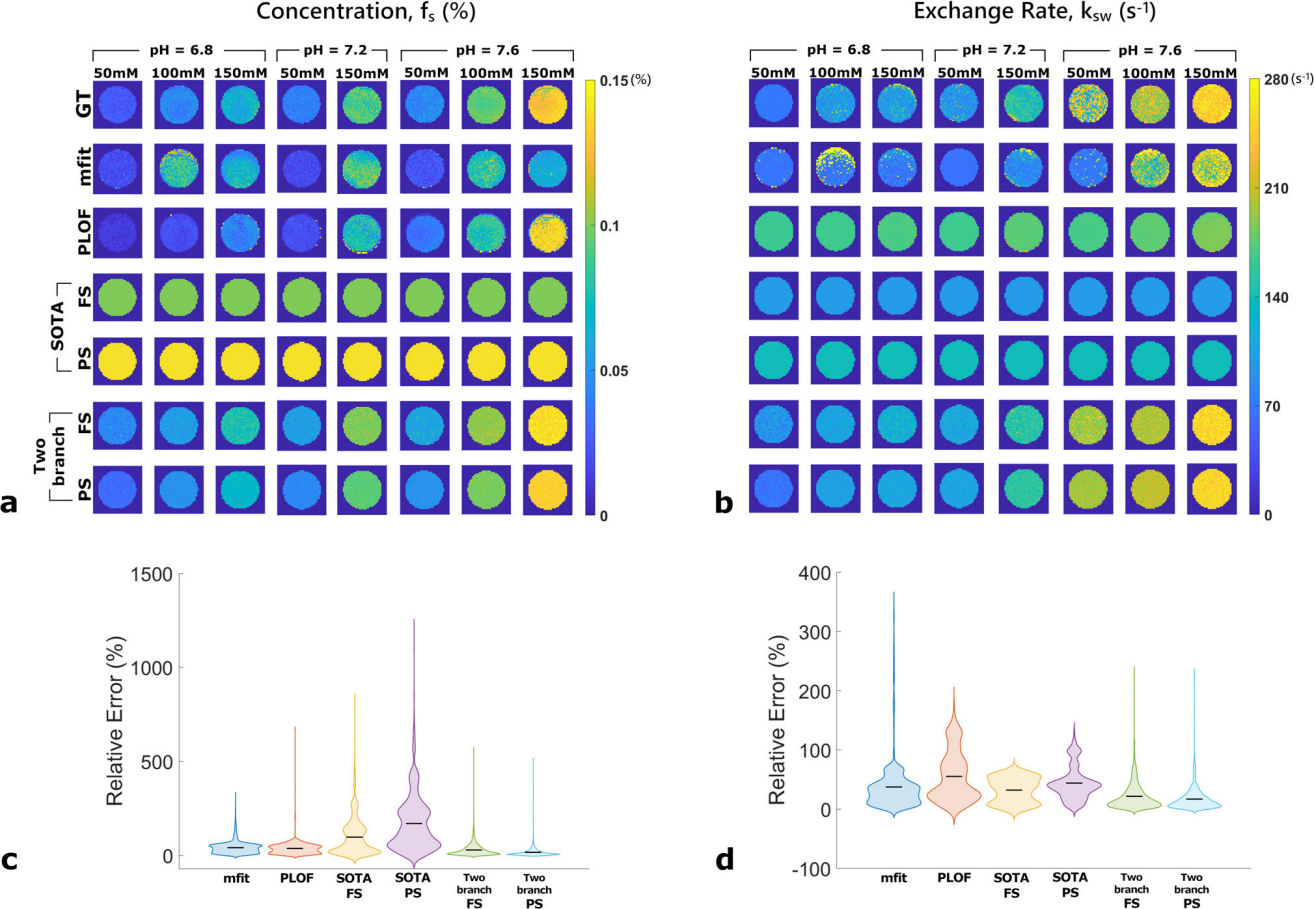
(a, b) Physical phantoms displaying ground truth and predicted *f*_s_ and *k*_sw_ maps respectively, with an approximate noise level of 40 dB, obtained using six various quantification methods. (c, d) Corresponding violin plots of the relative errors for *f*_s_ and *k*_sw_ respectively. The six methods are multi‐pool Lorentzian fitting (mfit), polynomial and Lorentzian line‐shape fitting (PLOF), the *state‐of‐the‐art* (SOTA) model trained on fully synthetic (SOTA FS) and partially synthetic (SOTA PS) data, the proposed two‐branch global–local model trained on fully synthetic (Two branch FS) and partially synthetic (Two branch PS) data. The estimated SNR of the experimental acquisitions was approximately 40 dB.

Figure 5 presents a comparison of the performance of various methods for quantifying *f*_s_ and *k*_sw_ in the physical phantoms. Figure 5a,c show the ground truth along with the quantified phantom maps and their corresponding relative error maps for *f*_s_, while Figure 5b,d present the same for *k*_sw_. The estimated SNR of the experimental acquisitions was approximately 40 dB.

Across all eight phantoms the Two‐branch PS model demonstrates the lowest MRE values and the most spatially homogeneous maps for both parameters with values of 14.89% ± 18.75% for *f*_s_ and 17.15% ± 18.20% for *k*_sw_, outperforming all other approaches. In contrast, traditional fitting methods exhibit higher errors. Specifically, mfit yields MRE values of 41.04% ± 32.64% for *f*_s_ and 37.38% ± 41.79% for *k*_sw_, while PLOF results in 36.16% ± 29.64% and 54.92% ± 41.37%, respectively. The Two‐branch FS model performs better than the traditional fitting methods with MRE values of 29.01% ± 31.72% for *f*_s_ and 21.26% ± 21.73% for *k*_sw_; however, its errors remain higher than those of the Two‐branch PS model. The SOTA FS and SOTA PS models show the highest MRE among learning‐based approaches. Specifically, the SOTA FS model yields MRE values of 96.92% ± 96.66% for *f*_s_ and 32.38% ± 20.51% for *k*_sw_, while the SOTA PS model results in 168.67% ± 142.03% and 43.70% ± 27.61%, respectively. Notably, their predicted *f*_s_ and *k*_sw_ values remain nearly constant across all eight phantoms, demonstrating minimal sensitivity to variations in concentration and pH. A clear concentration‐ and pH‐dependent trend is observed in the ground truth. The *f*_s_ increases approximately linearly with concentration (50–150 mM), while *k*_sw_ exhibits an exponential increase with pH. The Two‐branch FS and PS models accurately preserve these expected trends, whereas other methods show flattened or random responses.

### 3.4 Comparison Study of Various Quantification Methods in Animal Model

**FIGURE 6:**
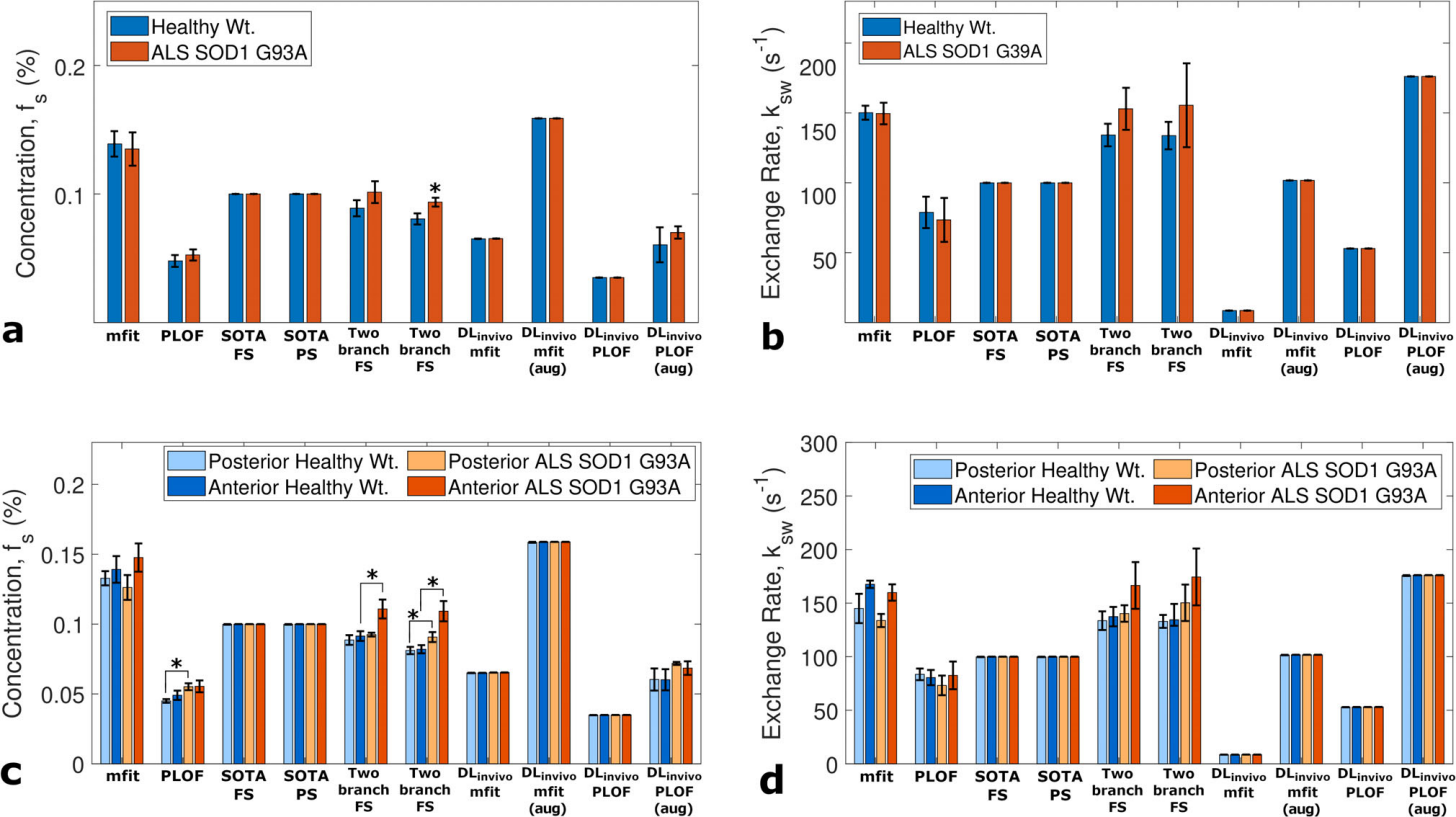
Statistical comparisons of (a) *f*_s_ and (b) *k*_sw_ quantified by ten different methods between all healthy Wt rats (blue) and ALS rats (orange) from whole muscle ROI. Regional differences in posterior and anterior regions for *f*_s_ (c) and *k*_sw_ (d) across ten different methods. The ten methods are multi‐pool Lorentzian fitting (mfit), polynomial and Lorentzian line‐shape fitting (PLOF), the *state‐of‐the‐art* (SOTA) model trained on fully synthetic (SOTA FS) and partially synthetic (SOTA PS) data, the proposed two‐branch global–local model trained on fully synthetic (Two branch FS) and partially synthetic (Two branch PS) data, model trained using measured in vivo data with mfit or PLOF targets (DL_invivo_ mfit and DL_invivo_ PLOF), each trained either without augmentation or with data augmentation (aug). *p < 0.05.

Figure 6 presents statistical differences of the quantified or predicted PCr *f*_s_ and *k*_sw_ values between all healthy Wt rats and ALS rats and across various regions for various methods. The Two‐branch PS model demonstrated a statistically significant difference for f_s_ (*p* = 0.0147) while none of the other methods exhibit statistically significant separation for either parameter. The lack of significant separation for the models trained on in vivo data (DL_invivo_ mfit, DL_invivo_ PLOF, DL_invivo_ mfit (aug), DL_invivo_ PLOF (aug)) may be attributed to the intrinsically inaccurate or noisy training targets and the limited sample size. Regional analysis of the posterior and anterior compartments revealed differences in f_s_ using PLOF and the Two‐branch PS and Two‐branch FS models. The Two‐branch PS model was the only one that exhibited statistical significance in both the posterior and anterior regions. The Two‐branch FS model showed statistical significance in the anterior regions while the PLOF method showed significant changes in the posterior regions. The remaining methods did not demonstrate statistically significant regional differences in *f*_s_. No significant differences in *k*_sw_ were observed between control and ALS animals in either posterior or anterior muscle groups across any of the evaluated methods.

**FIGURE 7:**
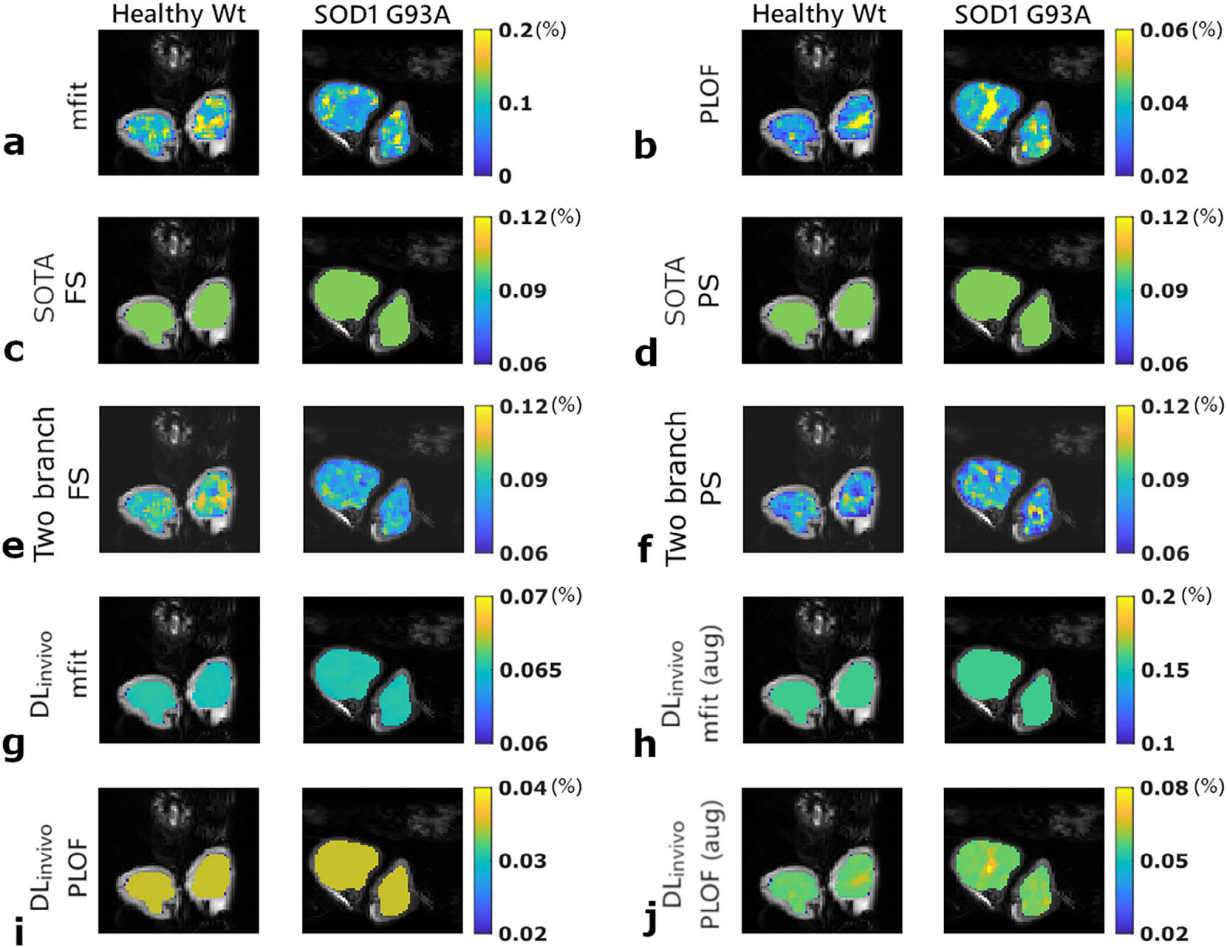
Maps of f_s_ quantified by ten different methods in a‐j on a representative healthy Wt rat leg and a representative SOD1 G93A ALS rat leg. The ten methods are multi‐pool Lorentzian fitting (mfit), polynomial and Lorentzian line‐shape fitting (PLOF), the *state‐of‐the‐art* (SOTA) model trained on fully synthetic (SOTA FS) and partially synthetic (SOTA PS) data, the proposed two‐branch global–local model trained on fully synthetic (Two branch FS) and partially synthetic (Two branch PS) data, a model trained using measured in vivo data with mfit or PLOF targets (DL_invivo_ mfit and DL_invivo_ PLOF), each trained either without augmentation or with data augmentation (aug). The estimated SNR of the experimental acquisitions was approximately 33.98 dB.

**FIGURE 8:**
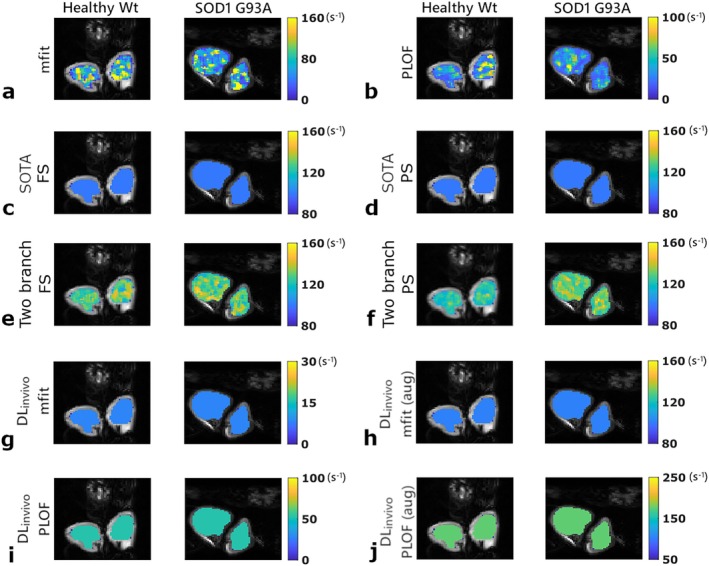
Maps of k_sw_ quantified by ten different methods in a‐j on a representative healthy Wt rat leg and a representative SOD1 G93A ALS rat leg. The ten methods are multi‐pool Lorentzian fitting (mfit), polynomial and Lorentzian line‐shape fitting (PLOF), the *state‐of‐the‐art* (SOTA) model trained on fully synthetic (SOTA FS) and partially synthetic (SOTA PS) data, the proposed two‐branch global–local model trained on fully synthetic (Two branch FS) and partially synthetic (Two branch PS) data, model trained using measured in vivo data with mfit or PLOF targets (DL_invivo_ mfit and DL_invivo_ PLOF), each trained either without augmentation or with data augmentation (aug). The estimated SNR of the experimental acquisitions was approximately 33.98 dB.

Figures 7 and 8 display the maps of the quantified or predicted PCr *f*_s_ and *k*_sw_, respectively, in the skeletal muscle of a representative healthy Wt rat leg and a representative ALS rat leg. The estimated SNR of the experimental acquisitions was approximately 33.98 dB. Two‐branch FS, Two‐branch PS, mfit, and PLOF were the only methods that demonstrated clear spatial variation in PCr *f*_s_ and *k*_sw_ maps within muscle tissue. In contrast, the *SOTA* models show nearly no spatial variation. This behavior is consistent with results from the digital and physical phantoms and suggests that the simple *SOTA* model fails to capture the relationship between the Z‐spectra and the underlying PCr parameters. Additionally, the model trained using measured in vivo data with targets derived from mfit or PLOF fails to produce meaningful contrast. This lack of contrast may be attributed to insufficient diversity in the in vivo dataset which constrains the model's ability to learn physiologically relevant variations.

### 3.5 Repeatability Study

**FIGURE 9:**
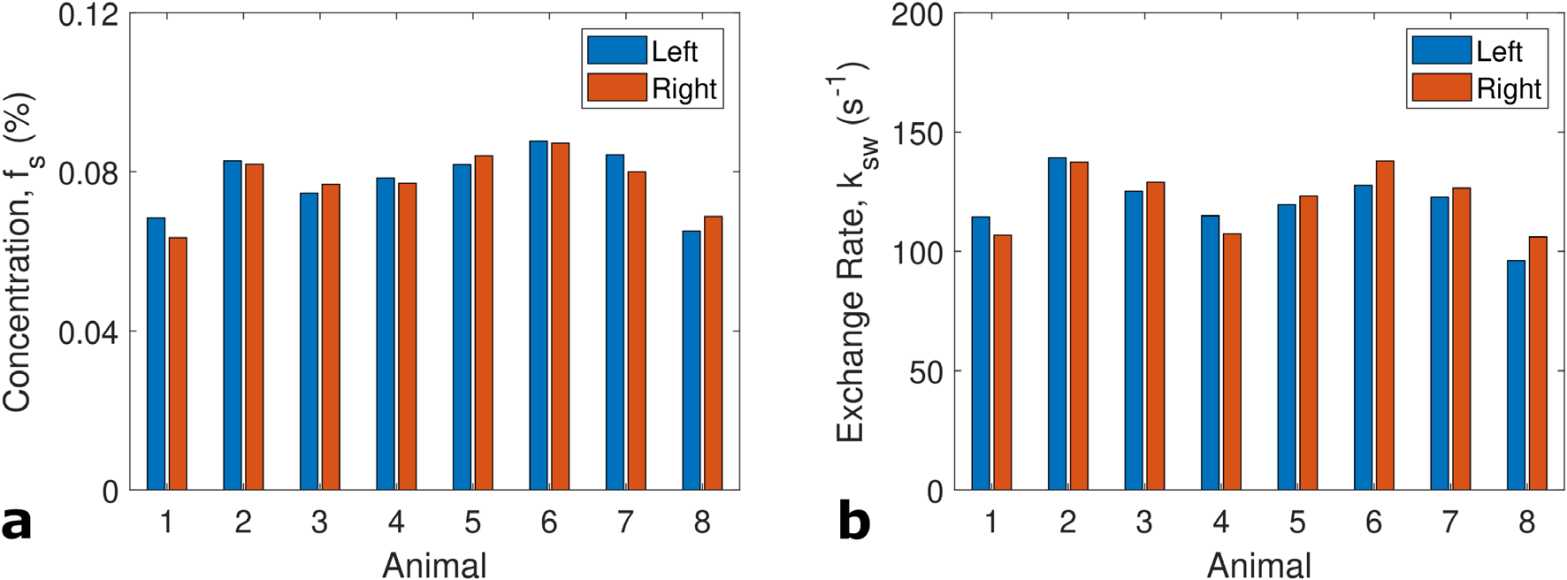
Quantified *f*_s_ and *k*_sw_ across eight Wt animals using the two‐branch model trained using partially synthetic data. The relative changes in f_s_ between left (blue) and right (orange) legs are 5.71%, 1.02%, −2.57%, 1.47%, −2.57%, 0.60%, 4.96%, and −4.16% for the eight animals respectively. The relative changes in k_sw_ between left and right legs are 5.62%, 1.23%, −2.76%, 5.49%, −2.66%, −7.46%, −2.74%, and −7.18% for the eight animals respectively.

Figure 9 shows the quantified *f*_s_ and *k*_sw_ by the proposed method. Across the eight animals, the coefficient of variation (CV) was approximately 10.17% and 10.19% for *f*_s_ in the right and left legs, respectively. For *k*_sw_, the CV across animals was approximately 10.37% in the right leg and 11.03% in the left leg. The mean absolute relative difference between left and right legs within animals was 2.88% for *f*_s_ and 4.39% for *k*_sw_. The maximum observed bilateral absolute relative difference was 5.71% for *f*_s_ observed in animal #1 and 7.46% for k_sw_ observed in animal #6.

## 4 Discussion

This study developed a global–local two‐branch DL model to predict the PCr *f*_s_ and *k*_sw_ in CEST MRI. Compared with the traditional mfit and PLOF methods as well as the *SOTA* (ANNCEST) DL model, our proposed method demonstrates significantly enhanced accuracy and robustness to imaging noises, performing efficiently even under low SNR conditions. This improvement is achieved through the combined use of a global branch to capture background confounding effects and noise distribution, a local branch to detect fine changes in PCr f_s_ and *k*_sw_, and the use of partially synthetic data for effective training. In contrast, the *SOTA* model employs a conventional fully connected architecture, which may not effectively capture these variations in the Z‐spectrum. Furthermore, its reliance on fully synthetic data for training as well as shortened Z‐spectrum using a single power may limit its ability to accurately learn real tissue characteristics. Hence, previous studies indicated that it requires a very high SNR with gaussian white noise of standard deviation 0.0035 to achieve optimal performance [15].

Additionally, the mfit method may struggle to resolve overlapping pools at low magnetic fields, while PLOF requires manual selection of polynomial order and fitting regions, which can introduce bias in quantification.

Our experiments showed that PCr *f*_s_ was 0.0845% ± 0.0216% in healthy Wt. Muscle and 0.0936% ± 0.018% in ALS muscle. Because the PCr CEST signal at 2.6 ppm originates from the –NH_2_ group containing two exchangeable protons, the estimated PCr concentration was 46.48 ± 11.88 mM in healthy muscle and 51.48 ± 9.90 mM in ALS muscle. The PCr concentration in healthy rat muscle was found to be 25.18 ± 7.36 mM and 38.30 ± 4.44 mM in ALS muscle using ^31^P MRS. Muscle tissue contains approximately 70%–75% water [50], which can be further categorized into tightly bound water (15%), loosely bound water (60%), and free water (25%) [51]. Assuming 70% of water contributes to the CEST signals, our calculated PCr concentration in healthy muscle could be approximately 27.66 ± 7.07 (46.48 ± 11.88 mM × 0.7 × (0.60 + 0.25) = 27.66 ± 7.07 mM) mmol (l tissue)^−1^. The increased PCr concentration in ALS muscle was found to be around 30.63 ± 5.89 (51.48 ± 9.90 × 0.7 × (0.6 + 0.25) = 30.63 ± 5.89 mM) mmol (l tissue)^−1^. This increase may reflect pathological remodeling in ALS skeletal muscle, supported by H&E staining in Figure S1 showing nuclear infiltration and centralized nuclei. Figure S2 further substantiates this observation, demonstrating elevated PCr amplitude in ALS skeletal muscle, consistent with increased PCr *f*_s_, and a strong correlation with the two metrics (*r* = 0.886 with *p* = 0.0333). Additionally, the PCr *k*_sw_ is 133.78 ± 9.81 s^−1^ in healthy muscle and 155.49 ± 30.01 s^−1^ in ALS muscle. Although the PCr *k*_sw_ was elevated in ALS skeletal muscle, there was a lack of statistical significance.

Although this study focuses on a preclinical rat skeletal muscle model, the proposed framework has strong translational potential for human imaging. The proposed model was evaluated at 4.7 T, a field strength closer to clinical scanners, and was designed to remain robust under low SNR and overlapping CEST effects commonly encountered in human imaging. Moreover, the anatomical structure, muscle fiber organization, and biochemical processes, namely CK mediated energy transfer and pH‐sensitive exchange, are conserved between rodents and humans. Therefore, the approach can be directly adapted to clinical field strengths of 3 T or 7 T.

One limitation of our proposed method is the extended scan duration, as it necessitates obtaining two complete Z‐spectra with varying B_1_ values. In translation to clinical human MRI applications, where B_1_ inhomogeneity is more significant, it may be necessary to measure and compensate for B_1_ shifts which may further extend the scan time. Future research should focus on optimizing the number of acquisition points or using rapid acquisition techniques to reduce scan time effectively [52, 53, 54]. Furthermore, this study focuses on detecting metabolic alterations at an early stage of ALS. Due to the tightly controlled genetic background and reproducible disease progression of the SOD1 G93A model [55], the observed histological features were consistent across the animals, allowing reliable interpretation even with a small cohort. Future longitudinal studies with larger cohorts across progressive disease stages will allow a more comprehensive characterization of *f*_s_ and *k*_sw_ evolution and their relationship with tissue pathology.

## 5 Conclusions

In conclusion, the development of this advanced DL model allows for accurate and robust quantification of the PCr concentration and tissue pH (related to *k*_sw_) in vivo muscle tissues. Importantly, our model can detect minute changes in PCr *f*_s_ and *k*_sw_, identifying metabolic alterations even in presymptomatic ALS, unlike conventional fitting approaches and existing state‐of‐the‐art DL models.

## Funding

This work was supported by National Institutes of Health (R01NS140757, R01EB036574, R01EB029443, R21AG089699).

## Supporting information


**Table S1:** Initial and boundary parameters for the amplitude (A), width (W), and offset (Δ) of all pools in the six‐pool model Lorentzian fit. The unit of peak width and offset is ppm.
**Table S2:** Initial and boundary parameters for the polynomial coefficients.
**Table S3:** Sample parameters used to generate partially synthetic data with measured components from digital phantoms or in vivo data.
**Table S4:** Sample parameters used to generate partially synthetic data with measured components from physical phantoms.
**Table S5:** Sample parameters used to generate fully synthetic data.
**Table S6:** Training details of the proposed DL model.
**Table S7:** Sample parameters used to generate the digital phantom data.
**Table S8:** Initial and boundary parameters for the amplitude (A), width (W), and offset (Δ) of all pools in the three‐pool model Lorentzian fit for the PCr only ground truth phantoms. The unit of peak width and offset is ppm.
**Figure S1:** Validation of animal model with histological evidence and blood serum CK. To provide complementary non‐MR validation, histological staining and blood analyses were performed at the Vanderbilt University Medical Center Translation Pathology Shared Resource (VUMC TPSR). Skeletal muscle samples were collected following imaging, fixed in 10% neutral‐buffered formalin, paraffin‐embedded, sectioned into 5 μm slices, and processed for hematoxylin and eosin (H&E) staining to assess muscle morphology and structural integrity. Blood samples were collected by TPSR and sent to Antech GLP lab for serum biochemical analysis, including CK. H&E‐stained sections were digitally scanned at the Vanderbilt University Medical Center Digital Histology Shared Resource, and whole‐slide images were used for visualization and analysis. (a) H and E staining in Healthy Wt. Muscle with packed bundle fibers with evenly distributed nuclei around the fiber. (b) H and E staining in SOD1‐G93A muscle shows variability in fiber size and increased nuclear infiltration, with clustered and centrally located nuclei (*) indicating muscle fiber degeneration and remodeling.
**Figure S2:** (a) ^31^P‐MRS spectra from ALS skeletal muscle (red) and control Wt. Skeletal muscle (blue). (b) Correlation between the ^31^P‐MRS detected PCr concentration and the predicted PCr concentration using our proposed two‐branch PS method was found to be *r* = 0.886 with *p* = 0.0333. ^31^P MRS experiments were performed on 9.4 T Bruker Biospec system. For animal positioning and anatomical reference imaging, a 63 mm quadrature volume coil was used to acquire proton images. For ^31^P, a custom‐built surface coil tuned to 162 MHz, corresponding to the resonant frequency of ^31^P at 9.4 T was used. The surface coil was placed directly over the hindlimb muscle. A small vial filled with phosphate‐buffered saline (PBS) was positioned adjacent to the coil to facilitate localization for shimming and slice selection. Spectra were acquired using the image‐selected in vivo spectroscopy (ISIS) technique with a voxel size 10 × 6 × 5 mm^3^ covering the entire muscle region of interest. TR was set to 4000 ms, and a spectral bandwidth of ±10 ppm was used. A total of 144 ISIS averages were acquired. Prior to spectral acquisition, localized shimming was performed to optimize magnetic field homogeneity within the voxel. The PCr resonance was manually referenced to 0 ppm using the chemical shift obtained from the single‐pulse acquisition.

## Data Availability

All data and processing codes are available at lab's GitHub account, CESTLabZu under the repository name “DL_PCr_muscle.” https://github.com/CESTlabZu/DL_PCr_muscle.

## References

[1] L. Guimarães‐Ferreira , “Role of the Phosphocreatine System on Energetic Homeostasis in Skeletal and Cardiac Muscles,” Einstein 12, no. 1 (2014): 126–131. 24728259 10.1590/S1679-45082014RB2741 PMC4898252

[2] M. F. Baird , S. M. Graham , J. S. Baker , and G. F. Bickerstaff , “Creatine‐Kinase‐ and Exercise‐Related Muscle Damage Implications for Muscle Performance and Recovery,” Journal of Nutrition and Metabolism 2012 (2012): 960363. 22288008 10.1155/2012/960363 PMC3263635

[3] Z. Simmons , B. L. Peterlin , P. J. Boyer , and J. Towfighi , “Muscle Biopsy in the Evaluation of Patients With Modestly Elevated Creatine Kinase Levels,” Muscle & Nerve 27, no. 2 (2003): 242–244. 12548533 10.1002/mus.10292

[4] M. Viswanathan , Y. Kurmi , and Z. Zu , “A Rapid Method for Phosphocreatine‐Weighted Imaging in Muscle Using Double Saturation Power‐Chemical Exchange Saturation Transfer,” NMR in Biomedicine 37, no. 4 (2024): e5089. 38114069 10.1002/nbm.5089 PMC12459672

[5] K. M. Ward , A. H. Aletras , and R. S. Balaban , “A New Class of Contrast Agents for MRI Based on Proton Chemical Exchange Dependent Saturation Transfer (CEST),” Journal of Magnetic Resonance 143, no. 1 (2000): 79–87. 10698648 10.1006/jmre.1999.1956

[6] J. Y. Zhou and P. C. M. van Zijl , “Chemical Exchange Saturation Transfer Imaging and Spectroscopy,” Progress in Nuclear Magnetic Resonance Spectroscopy 48, no. 2–3 (2006): 109–136.

[7] P. C. M. van Zijl and N. N. Yadav , “Chemical Exchange Saturation Transfer (CEST): What Is in a Name and What Isn't?,” Magnetic Resonance in Medicine 65, no. 4 (2011): 927–948. 21337419 10.1002/mrm.22761 PMC3148076

[8] J. Kim , Y. Wu , Y. Guo , H. Zheng , and P. Z. Sun , “A Review of Optimization and Quantification Techniques for Chemical Exchange Saturation Transfer MRI Toward Sensitive In Vivo Imaging,” Contrast Media & Molecular Imaging 10, no. 3 (2015): 163–178. 25641791 10.1002/cmmi.1628 PMC4469549

[9] B. Wu , G. Warnock , M. Zaiss , et al., “An Overview of CEST MRI for Non‐MR Physicists,” EJNMMI Physics 3, no. 1 (2016): 19. 27562024 10.1186/s40658-016-0155-2 PMC4999387

[10] P. C. M. van Zijl , W. W. Lam , J. D. Xu , L. Knutsson , and G. J. Stanisz , “Magnetization Transfer Contrast and Chemical Exchange Saturation Transfer MRI. Features and Analysis of the Field‐Dependent Saturation Spectrum,” NeuroImage 168 (2018): 222–241. 28435103 10.1016/j.neuroimage.2017.04.045 PMC5650949

[11] G. Liu , X. Song , K. W. Chan , and M. T. McMahon , “Nuts and Bolts of Chemical Exchange Saturation Transfer MRI,” NMR in Biomedicine 26, no. 7 (2013): 810–828. 23303716 10.1002/nbm.2899 PMC4144273

[12] E. Vinogradov , “Imaging Molecules,” Journal of Magnetic Resonance 306 (2019): 145–149. 31337563 10.1016/j.jmr.2019.07.022

[13] J. J. Chung , T. Jin , J. H. Lee , and S.‐G. Kim , “Chemical Exchange Saturation Transfer Imaging of Phosphocreatine in the Muscle,” Magnetic Resonance in Medicine 81, no. 6 (2019): 3476–3487. 30687942 10.1002/mrm.27655 PMC6435392

[14] L. Chen , P. B. Barker , R. G. Weiss , P. C. M. van Zijl , and J. D. Xu , “Creatine and Phosphocreatine Mapping of Mouse Skeletal Muscle by a Polynomial and Lorentzian Line‐Shape Fitting CEST Method,” Magnetic Resonance in Medicine 81, no. 1 (2019): 69–78. 30246265 10.1002/mrm.27514 PMC6258268

[15] L. Chen , M. Schär , K. W. Y. Chan , et al., “In Vivo Imaging of Phosphocreatine With Artificial Neural Networks,” Nature Communications 11, no. 1 (2020): 1072. 10.1038/s41467-020-14874-0 PMC7044432 32102999

[16] L. Ju , K. Wang , M. Schar , et al., “Simultaneous Creatine and Phosphocreatine Mapping of Skeletal Muscle by CEST MRI at 3T,” Magnetic Resonance in Medicine 91, no. 3 (2024): 942–954. 37899691 10.1002/mrm.29907 PMC10842434

[17] J. D. Xu , J. J. Chung , and T. Jin , “Chemical Exchange Saturation Transfer Imaging of Creatine, Phosphocreatine, and Protein Arginine Residue in Tissues,” NMR in Biomedicine 36, no. 6 (2023): e4671. 34978371 10.1002/nbm.4671 PMC9250548

[18] K. Wang , J. Huang , L. Ju , et al., “Creatine Mapping of the Brain at 3T by CEST MRI,” Magnetic Resonance in Medicine 91, no. 1 (2024): 51–60. 37814487 10.1002/mrm.29876 PMC10843037

[19] Z. Q. Zhang , K. X. Wang , S. Park , et al., “The Exchange Rate of Creatine CEST in Mouse Brain,” Magnetic Resonance in Medicine 90 (2023): 373–384. 37036030 10.1002/mrm.29662 PMC11054327

[20] K. Wang , S. Park , D. O. Kamson , Y. Li , G. Liu , and J. Xu , “Guanidinium and Amide CEST Mapping of Human Brain by High Spectral Resolution CEST at 3 T,” Magnetic Resonance in Medicine 89, no. 1 (2023): 177–191. 36063502 10.1002/mrm.29440 PMC9617768

[21] L. Chen , Z. Wei , S. Cai , et al., “High‐Resolution Creatine Mapping of Mouse Brain at 11.7 T Using Non‐Steady‐State Chemical Exchange Saturation Transfer,” NMR in Biomedicine 32, no. 11 (2019): e4168. 31461196 10.1002/nbm.4168

[22] O. Cohen , V. Y. Yu , K. R. Tringale , et al., “CEST MR Fingerprinting (CEST‐MRF) for Brain Tumor Quantification Using EPI Readout and Deep Learning Reconstruction,” Magnetic Resonance in Medicine 89, no. 1 (2023): 233–249. 36128888 10.1002/mrm.29448 PMC9617776

[23] J. P. Huang , J. H. C. Lai , K. H. Tse , et al., “Deep Neural Network Based CEST and AREX Processing: Application in Imaging a Model of Alzheimer's Disease at 3 T,” Magnetic Resonance in Medicine 87, no. 3 (2022): 1529–1545. 34657318 10.1002/mrm.29044

[24] L. Hunger , J. R. Rajput , K. Klein , et al., “DeepCEST 7 T: Fast and Homogeneous Mapping of 7 T CEST MRI Parameters and Their Uncertainty Quantification,” Magnetic Resonance in Medicine 89, no. 4 (2023): 1543–1556. 36377762 10.1002/mrm.29520

[25] G. Karunanithy , T. Yuwen , L. E. Kay , and D. F. Hansen , “Towards Autonomous Analysis of Chemical Exchange Saturation Transfer Experiments Using Deep Neural Networks,” Journal of Biomolecular NMR 76, no. 3 (2022): 75–86. 35622310 10.1007/s10858-022-00395-z PMC9246985

[26] B. Kim , M. Schar , H. Park , and H. Y. Heo , “A Deep Learning Approach for Magnetization Transfer Contrast MR Fingerprinting and Chemical Exchange Saturation Transfer Imaging,” NeuroImage 221 (2020): 117165. 32679254 10.1016/j.neuroimage.2020.117165

[27] S. Mohammed Ali , N. N. Yadav , R. Wirestam , et al., “Deep Learning‐Based Lorentzian Fitting of Water Saturation Shift Referencing Spectra in MRI,” Magnetic Resonance in Medicine 90, no. 4 (2023): 1610–1624. 37279008 10.1002/mrm.29718 PMC10524193

[28] O. Perlman , H. Ito , K. Herz , et al., “Quantitative Imaging of Apoptosis Following Oncolytic Virotherapy by Magnetic Resonance Fingerprinting Aided by Deep Learning,” Nature Biomedical Engineering 6, no. 5 (2022): 648–657. 10.1038/s41551-021-00809-7 PMC9091056 34764440

[29] M. Singh , S. Jiang , Y. Li , P. van Zijl , J. Zhou , and H. Y. Heo , “Bloch Simulator‐Driven Deep Recurrent Neural Network for Magnetization Transfer Contrast MR Fingerprinting and CEST Imaging,” Magnetic Resonance in Medicine 90, no. 4 (2023): 1518–1536. 37317675 10.1002/mrm.29748 PMC10524222

[30] W. Yang , J. Zou , X. Zhang , Y. Chen , H. Tang , and G. Xiao , “An End‐To‐End LSTM‐Attention Based Framework for Quasi‐Steady‐State CEST Prediction,” Frontiers in Neuroscience 17 (2023): 1281809. 38249583 10.3389/fnins.2023.1281809 PMC10797904

[31] F. Glang , A. Deshmane , S. Prokudin , et al., “DeepCEST 3T: Robust MRI Parameter Determination and Uncertainty Quantification With Neural Networks‐Application to CEST Imaging of the Human Brain at 3T,” Magnetic Resonance in Medicine 84, no. 1 (2020): 450–466. 31821616 10.1002/mrm.28117

[32] M. Zaiss , A. Deshmane , M. Schuppert , et al., “DeepCEST: 9.4 T Chemical Exchange Saturation Transfer MRI Contrast Predicted From 3 T Data‐A Proof of Concept Study,” Magnetic Resonance in Medicine 81, no. 6 (2019): 3901–3914. 30803000 10.1002/mrm.27690

[33] S. Q. Pan , Y. C. Hum , K. W. Lai , et al., “Artificial Intelligence in Chemical Exchange Saturation Transfer Magnetic Resonance Imaging,” Artificial Intelligence Review 58, no. 7 (2025): 210.

[34] J. Wang , P. Cai , Z. Wang , H. Zhang , and J. Huang , “CEST MRI Data Analysis Using Kolmogorov‐Arnold Network (KAN) and Lorentzian‐KAN (LKAN) Models,” Magnetic Resonance in Medicine 94 (2025): 1301–1317. 40468586 10.1002/mrm.30548 PMC12202730

[35] Y. Li , D. Xie , A. Cember , et al., “Accelerating GluCEST Imaging Using Deep Learning for B0 Correction,” Magnetic Resonance in Medicine 84, no. 4 (2020): 1724–1733. 32301185 10.1002/mrm.28289 PMC8082274

[36] C. Shen , K. Cheema , Y. Xie , D. Ruan , and D. Li , “Accelerating CEST MRI With Deep Learning‐Based Frequency Selection and Parameter Estimation,” NMR in Biomedicine 38, no. 7 (2025): e70068. 40396230 10.1002/nbm.70068

[37] K. Cheema , R. De Righi Dante , C. Shen , et al., “Accelerated 3D qCEST of the Spine in a Porcine Model Using MR Multitasking at 3T,” NMR in Biomedicine 38, no. 9 (2025): e70122. 40817426 10.1002/nbm.70122

[38] M. Viswanathan , L. Yin , Y. Kurmi , et al., “A Rapid and Accurate Guanidine CEST Imaging in Ischemic Stroke Using a Machine Learning Approach,” Physics in Medicine and Biology 71, no. 4 (2026): 045006. 10.1088/1361-6560/ae4167 PMC12914501 41633047

[39] L. Yin , M. Viswanathan , Y. Kurmi , et al., “Improving Quantification Accuracy of a Nuclear Overhauser Enhancement Signal at −1.6 Ppm at 4.7 T Using a Machine Learning Approach,” Physics in Medicine and Biology 70, no. 2 (2025): 025009. 10.1088/1361-6560/ada716 PMC11740009 39774035

[40] Y. Kurmi , M. Viswanathan , L. Yin , et al., “Denoising Low‐Power CEST Imaging Using a Deep Learning Approach With a Dual‐Power Feature Preparation Strategy,” Magnetic Resonance in Medicine 95, no. 3 (2026): 1410–1428. 41082398 10.1002/mrm.70124 PMC12746379

[41] M. Viswanathan , L. Yin , Y. Kurmi , A. Afzal , and Z. Zu , “Enhancing Amide Proton Transfer Imaging in Ischemic Stroke Using a Machine Learning Approach With Partially Synthetic Data,” NMR in Biomedicine 38, no. 1 (2025): e5277. 39434444 10.1002/nbm.5277 PMC11602689

[42] M. Viswanathan , L. Yin , Y. Kurmi , and Z. Zu , “Machine Learning‐Based Amide Proton Transfer Imaging Using Partially Synthetic Training Data,” Magnetic Resonance in Medicine 91, no. 5 (2024): 1908–1922. 38098340 10.1002/mrm.29970 PMC10955622

[43] D. F. Gochberg and J. C. Gore , “Quantitative Magnetization Transfer Imaging via Selective Inversion Recovery With Short Repetition Times,” Magnetic Resonance in Medicine 57, no. 2 (2007): 437–441. 17260381 10.1002/mrm.21143 PMC2634834

[44] M. Viswanathan , Y. Kurmi , X. Jiang , J. Xu , and Z. Zu , “Interpreting Amide Proton Transfer‐Weighted Imaging Contrast Between Normal and Tumor Brain Tissues Using the Asymmetry Analysis Method at 4.7 T,” Magnetic Resonance in Medicine 95, no. 1 (2026): 485–505. 40851330 10.1002/mrm.70041 PMC12620183

[45] M. Zaiss , Z. L. Zu , J. Z. Xu , et al., “A Combined Analytical Solution for Chemical Exchange Saturation Transfer and Semi‐Solid Magnetization Transfer,” NMR in Biomedicine 28, no. 2 (2015): 217–230. 25504828 10.1002/nbm.3237 PMC4297271

[46] M. Zaiss and P. Bachert , “Chemical Exchange Saturation Transfer (CEST) and MR Z‐Spectroscopy In Vivo: A Review of Theoretical Approaches and Methods,” Physics in Medicine and Biology 58, no. 22 (2013): R221–R269. 24201125 10.1088/0031-9155/58/22/R221

[47] J. Cui , C. Sun , and Z. L. Zu , “NOE‐Weighted Imaging in Tumors Using Low‐Duty‐Cycle 2 Pi‐CEST,” Magnetic Resonance in Medicine 89, no. 2 (2023): 636–651. 36198015 10.1002/mrm.29475 PMC9792266

[48] Y. Zhao , C. S. Sun , and Z. L. Zu , “Isolation of Amide Proton Transfer Effect and Relayed Nuclear Overhauser Enhancement Effect At‐3.5ppm Using CEST With Double Saturation Powers,” Magnetic Resonance in Medicine 90, no. 3 (2023): 1025–1040. 37154382 10.1002/mrm.29691 PMC10646838

[49] Y. Kurmi , M. Viswanathan , and Z. Zu , “Enhancing SNR in CEST Imaging: A Deep Learning Approach With a Denoising Convolutional Autoencoder,” Magnetic Resonance in Medicine 92, no. 6 (2024): 2404–2419. 39030953 10.1002/mrm.30228

[50] I. Lorenzo , M. Serra‐Prat , J. C. Yébenes , I. Lorenzo , M. Serra‐Prat , and J. C. Yébenes , “The Role of Water Homeostasis in Muscle Function and Frailty: A Review,” Nutrients 11 (2019): 1857. 31405072 10.3390/nu11081857 PMC6723611

[51] E. Berényi , Z. Szendrö , P. Rózsahegyi , et al., “Postnatal Changes in Water Content and Proton Magnetic Resonance Relaxation Times in Newborn Rabbit Tissues,” Pediatric Research 39, no. 6 (1996): 1091–1098. 8725275 10.1203/00006450-199606000-00026

[52] J. Xu , T. Zu , Y. C. Hsu , X. Wang , K. W. Chan , and Y. Zhang , “DEISM: Deep Reconstruction Framework With Self‐Calibration Mechanisms for Accelerated Chemical Exchange Saturation Transfer Imaging,” in IEEE Transactions on Bio‐Medical Engineering (IEEE, 2025). 10.1109/TBME.2025.3543403 40036441

[53] J. Xu , T. Zu , Y. C. Hsu , X. Wang , K. W. Chan , and Y. Zhang , “Accelerating CEST Imaging Using a Model‐Based Deep Neural Network With Synthetic Training Data,” Magnetic Resonance in Medicine 91, no. 2 (2024): 583–599. 37867413 10.1002/mrm.29889

[54] T. Zu , X. Yong , Z. Dai , et al., “Prospective Acceleration of Whole‐Brain CEST Imaging by Golden‐Angle View Ordering in Cartesian Coordinates and Joint k‐Space and Image‐Space Parallel Imaging (KIPI),” Magnetic Resonance in Medicine 93, no. 4 (2025): 1585–1601. 39607875 10.1002/mrm.30375

[55] J. Stephenson and S. Amor , “Modelling Amyotrophic Lateral Sclerosis in Mice,” in Drug Discovery Today: Disease Models (Elsevier Ltd, 2017), 25–26.

