# Supplementary material for: Enhanced Quantitative Phosphocreatine MR Imaging of Skeletal Muscle Using a Global–Local Two‐Branch Deep Learning Model
Source: Magn Reson Med. 2026 Apr 10;96(2):741–55. doi: 10.1002/mrm.70386 (PMC13269197; doi:10.1002/mrm.70386)
Supplement: Supplementary file 1 — Table S1: Initial and boundary parameters for the amplitude (A), width (W), and offset (Δ) of all pools in the six‐pool model Lorentzian fit. The unit of peak width and offset is ppm. Table S2: Initial and boundary parameters for the polynomial coefficients. Table S3: Sample parameters used to generate partially synthetic data with measured components from digital phantoms or in vivo data. Table S4: Sample parameters used to generate partially synthetic data with measured components from physical phantoms. Table S5: Sample parameters used to generate fully synthetic data. Table S6: Training details of the proposed DL model. Table S7: Sample parameters used to generate the digital phantom data. Table S8: Initial and boundary parameters for the amplitude (A), width (W), and offset (Δ) of all pools in the three‐pool model Lorentzian fit for the PCr only ground truth phantoms. The unit of peak width and offset is ppm. Figure S1: Validation of animal model with histological evidence and blood serum CK. To provide complementary non‐MR validation, histological staining and blood analyses were performed at the Vanderbilt University Medical Center Translation Pathology Shared Resource (VUMC TPSR). Skeletal muscle samples were collected following imaging, fixed in 10% neutral‐buffered formalin, paraffin‐embedded, sectioned into 5 μm slices, and processed for hematoxylin and eosin (H&E) staining to assess muscle morphology and structural integrity. Blood samples were collected by TPSR and sent to Antech GLP lab for serum biochemical analysis, including CK. H&E‐stained sections were digitally scanned at the Vanderbilt University Medical Center Digital Histology Shared Resource, and whole‐slide images were used for visualization and analysis. (a) H and E staining in Healthy Wt. Muscle with packed bundle fibers with evenly distributed nuclei around the fiber. (b) H and E staining in SOD1‐G93A muscle shows variability in fiber size and increased nuclear infiltration, with clustere [file MRM-96-741-s001.docx]

**Supporting information**

**Supporting information Table S1.** Initial and boundary parameters for the amplitude (A), width (W), and offset (Δ) of all pools in the six-pool model Lorentzian fit. The unit of peak width and offset is ppm.

| Pool | Start | Lower | Upper | Pool | Start | Lower | Upper |
| --- | --- | --- | --- | --- | --- | --- | --- |
| A_water_ | 0.9 | 0.02 | 1 | A_amines/ guanidine_ | 0.01 | 0 | 0.2 |
| W_water_ | 1.4 | 0.1 | 10 | W_amines/guanidine_ | 1 | 0.5 | 2 |
| Δ_water_ | 0 | -1 | 1 | Δ_amines/ guanidine_ | 2 | 1.5 | 2.5 |
| A_amide_ | 0.025 | 0 | 0.2 | A_NOE_ | 0.02 | 0 | 1 |
| W_amide_ | 0.5 | 0.4 | 3 | W_NOE_ | 3 | 1 | 5 |
| Δ_amide_ | 3.5 | 3 | 4 | Δ_NOE_ | -3.5 | -4.5 | -2.5 |
| A_PCr_ | 0.01 | 0 | 0.2 | A_MT_ | 0.1 | 0 | 1 |
| W_PCr_ | 1 | 0.5 | 2.5 | W_MT_ | 25 | 10 | 100 |
| Δ_PCr_ | 2.6 | 2.5 | 3 | Δ_MT_ | 0 | -4 | 4 |

**Supporting information Table S2.** Initial and boundary parameters for the polynomial coefficients

|  | Start | Lower | Upper |
| --- | --- | --- | --- |
| C_0_ | 0.1 | 0 | 100 |
| C_1_ | 1 | 0 | 100 |
| C_2_ | 0.5 | 0 | 1000 |
| C_3_ | -190 | -1000 | 0 |

**Supporting information Table S3.** Sample parameters used to generate partially synthetic data with measured components from digital phantoms or *in vivo* data

| Pool | f_s_ (%) | k_sw_ (s^-1^) | T_1_ (s) | T_2_ (ms) | Δ (ppm) | r_measured_ |
| --- | --- | --- | --- | --- | --- | --- |
| water | 100 | - | 1.1:  0.3:  1.7 | 20:  20:  60 | 0 | - |
| amide | 0.08:  0.02:  0.12 | 100 | 1.5 | 2 | 3.6 | - |
| PCr | 0.06:  0.02:  0.14 | 60:  20:  160 | 1.5 | 8:2:12 | 2.6 |  |
| eGuanidine | 0.16:  0.04:  0.24 | 300:  200: 700 | 1.5 | 8:2:12 | 2 | - |
| NOE | 1.5 | 10 | 1.5 | 0.5 | -3.3 | - |
| amines | - | - | - | - | - | 0.75:  0.25:  1.25 |
| MT | - | - | - | - | - | 0.5:  0.5:  1.5 |

B_0_ shift applied with Δω_shift_ = -0.5:0.25:0.5ppm

B_1_ shift applied with β = 0.8:0.1:1.2

**Supporting information Table S4.** Sample parameters used to generate partially synthetic data with measured components from physical phantoms

| Pool | f_s_ (%) | k_sw_ (s^-1^) | T_1_ (s) | T_2_ (ms) | Δ (ppm) | r_measured_ |
| --- | --- | --- | --- | --- | --- | --- |
| water | 100 | - | 1.3:  0.5:  2.3 | 80:  20:  120 | 0 | - |
| amide | 0.05 | 100 | 1.5 | 2 | 3.6 | - |
| PCr | 0.08:  0.03:  0.20 | 60:  40:  180 | 1.5 | 10:2:14 | 2.6 |  |
| Guanidine | 0.1:  0.05:  0.2 | 300:  200: 700 | 1.5 | 10:2:14 | 2 | - |
| NOE | 1.5 | 10 | 1.5 | 0.5 | -3.3 | - |
| amines | - | - | - | - | - | 0.5:  0.5:  1.5 |
| MT | - | - | - | - | - | 0.8:  1:  1.2 |

B_0_ shift applied with Δω_shift_ = -0.5:0.25:0.5ppm

B_1_ shift applied with β = 0.8:0.1:1.2

**Supporting information Table S5.** Sample parameters used to generate fully synthetic data.

| Pool | f_s_ (%) | k_sw_ (s^-1^) | T_1_ (s) | T_2_ (ms) | Δ (ppm) |
| --- | --- | --- | --- | --- | --- |
| water | 100 | - | 1.1:  0.3:  1.7 | 20:  20:  60 | 0 |
| amide | 0.08:  0.02:  0.12 | 100 | 1.5 | 2 | 3.6 |
| PCr | 0.06:  0.02:  0.14 | 60:  20:  160 | 1.5 | 8:2:12 | 2.6 |
| Guanidine | 0.16:  0.04:  0.24 | 300:  200: 700 | 1.5 | 8:2:12 | 2 |
| NOE | 1.5 | 10 | 1.5 | 0.5 | -3.3 |
| amines | 0.18:  0.12:  0.42 | 3000:2000:  7000 | 1.5 | 15 | 3 |
| MT | 7.5:  7.5:  22.5 | 25 | 1.5 | 0.05 | -2.3 |

B_0_ shift applied with Δω_shift_ = -0.5:0.25:0.5ppm

B_1_ shift applied with β = 0.8:0.1:1.2

**Supporting Information Table S6.** Training details of the proposed DL model

| **Category** | **Parameter** | **Value** |
| --- | --- | --- |
| Framework | Library | PyTorch v2.5.1 |
| Hardware | GPU | NVIDIA RTX A5000 |
| Optimizer | Adam |  |
|  | Learning Rate | 1e-4 |
| Batch Size |  | 64 |
| Training epochs |  | 100 |
| Early stopping |  | 5 |
| Dataset splitting |  | 80:20 |
| Loss weights | $\lambda_{\mathrm{regress}}$ | 0.9 |
|  | $\lambda_{denoise}$ | 0.1 |
| Noise | Gaussian SD | 0.001-0.04 |
| Target scaling | f_s_ | X10000 |
|  | k_sw_ | /10 |
| Gradient optimization | Method | Projected Conflicting Gradient |
| Random seed |  | 42 |
| Training time | All simulated Datasets | ~5h |
|  | *in vivo* datasets | < 1 min |
|  | *in vivo* datasets with augmentation | ~1h |

**Supporting information Table S7.** Sample parameters used to generate the digital phantom data.

| Pool | f_s_ (%) | k_sw_ (s^-1^) | T_1_ (s) | T_2_ (ms) | Δ (ppm) |
| --- | --- | --- | --- | --- | --- |
| water | 100 | - | 1.2:0.2:  1.6 | 30:10:50 | 0 |
| amide | 0.1 | 100 | 1.5 | 2 | 3.6 |
| PCr | 0.08: 0.02:0.12 | 120:20:160 | 1.5 | 9:1:11 | 2.6 |
| Guanidine | 0.18: 0.02:0.22 | 300:200:  700 | 1.5 | 9:1:11 | 2 |
| NOE | 1.5 | 10 | 1.5 | 0.5 | -3.3 |
| amines | 0.24:  0.06:0.36 | 3000:  2000:  7000 | 1.5 | 15 | 3 |
| MT | 10:5:20 | 25 | 1.5 | 0.05 | -2.3 |

B_0_ shift applied with Δω_shift_ = -0.5:0:0.5ppm

B_1_ shift applied with β = 0.9:0.1:1.1

**Supporting information Table S8.** Initial and boundary parameters for the amplitude (A), width (W), and offset (Δ) of all pools in the three-pool model Lorentzian fit for the PCr only ground truth phantoms. The unit of peak width and offset is ppm.

| Pool | Start | Lower | Upper |
| --- | --- | --- | --- |
| A_water_ | 0.9 | 0.02 | 1 |
| W_water_ | 1.4 | 0.1 | 10 |
| Δ_water_ | 0 | -1 | 1 |
| A_PCr,2.6_ | 0.01 | 0 | 0.2 |
| W_PCr,2.6_ | 1 | 0.5 | 2.5 |
| Δ_PCr,2.6_ | 2.6 | 2.5 | 3 |
| A_PCr,2_ | 0.01 | 0 | 0.2 |
| W_PCr,2_ | 1 | 0.5 | 2 |
| Δ_PCr,2_ | 1.9 | 1.5 | 2.5 |

**
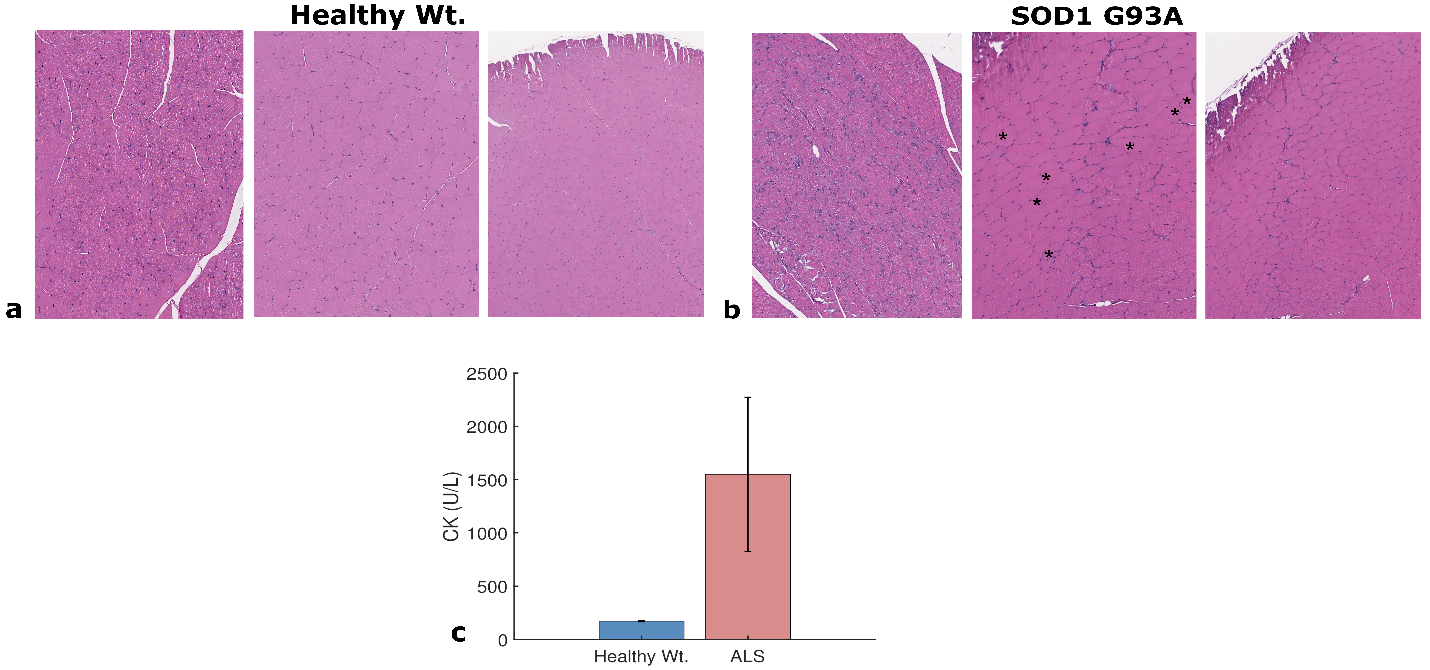
**

**Supporting Information Figure S1: Validation of animal model with histological evidence and blood serum CK.** To provide complementary non-MR validation, histological staining and blood analyses were performed at the Vanderbilt University Medical Center Translation Pathology Shared Resource (VUMC TPSR). Skeletal muscle samples were collected following imaging, fixed in 10% neutral-buffered formalin, paraffin-embedded, sectioned into 5 µm slices, and processed for hematoxylin and eosin (H&E) staining to assess muscle morphology and structural integrity. Blood samples were collected by TPSR and sent to Antech GLP lab for serum biochemical analysis, including CK. H&E-stained sections were digitally scanned at the Vanderbilt University Medical Center Digital Histology Shared Resource, and whole-slide images were used for visualization and analysis. (a) H and E staining in Healthy Wt. muscle with packed bundle fibers with evenly distributed nuclei around the fiber. (b) H and E staining in SOD1-G93A muscle shows variability in fiber size and increased nuclear infiltration, with clustered and centrally located nuclei (*) indicating muscle fiber degeneration and remodeling.


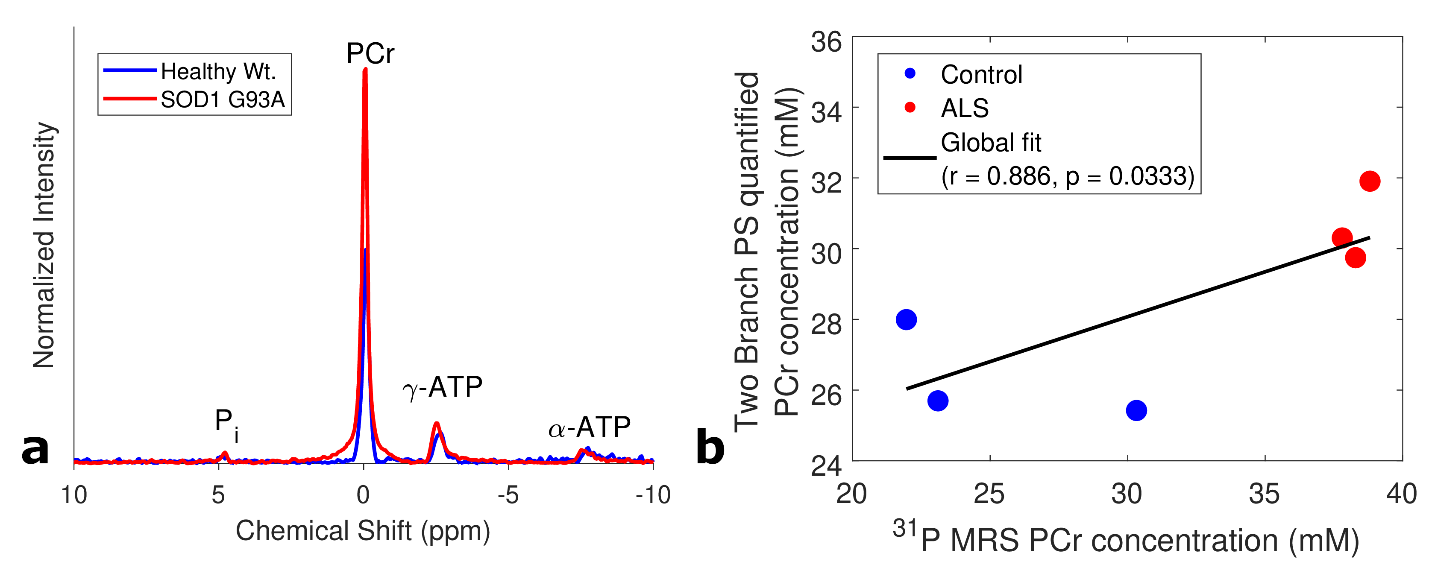


**Supporting Information Figure S2** (a) ^31^P-MRS spectra from ALS skeletal muscle (red) and control Wt. skeletal muscle (blue). (b) Correlation between the ^31^P-MRS detected PCr concentration and the predicted PCr concentration using our proposed two-branch PS method was found to be r = 0.886 with p = 0.0333. ^31^P MRS experiments were performed on 9.4T Bruker Biospec system. For animal positioning and anatomical reference imaging, a 63 mm quadrature volume coil was used to acquire proton images. For ^31^P, a custom-built surface coil tuned to 162MHz, corresponding to the resonant frequency of ^31^P at 9.4T was used. The surface coil was placed directly over the hindlimb muscle. A small vial filled with phosphate-buffered saline (PBS) was positioned adjacent to the coil to facilitate localization for shimming and slice selection. Spectra were acquired using the image-selected *in vivo* spectroscopy (ISIS) technique with a voxel size 10x6x5mm^3^ covering the entire muscle region of interest. TR was set to 4000ms, and a spectral bandwidth of ±10ppm was used. A total of 144 ISIS averages were acquired. Prior to spectral acquisition, localized shimming was performed to optimize magnetic field homogeneity within the voxel. The PCr resonance was manually referenced to 0 ppm using the chemical shift obtained from the single-pulse acquisition.
